# Genome-wide analysis of copy number variations identifies *PARK2* as a candidate gene for autism spectrum disorder

**DOI:** 10.1186/s13229-016-0087-7

**Published:** 2016-04-01

**Authors:** Chia-Lin Yin, Hsin-I Chen, Ling-Hui Li, Yi-Ling Chien, Hsiao-Mei Liao, Miao Chun Chou, Wen-Jiun Chou, Wen-Che Tsai, Yen-Nan Chiu, Yu-Yu Wu, Chen-Zen Lo, Jer-Yuarn Wu, Yuan-Tsong Chen, Susan Shur-Fen Gau

**Affiliations:** Department of Psychiatry, National Taiwan University Hospital and College of Medicine, No. 7, Chung-Shan South Road, Taipei, 10002 Taiwan; Graduate Institute of Brain Sciences, National Yang-Ming University, Taipei, 11221 Taiwan; Institute of Biomedical Sciences, Academia Sinica, Taipei, 11529 Taiwan; Section on Molecular Neurobiology, National Institute of Mental Health, National Institutes of Health, Bethesda, 20892 USA; Department of Child Psychiatry, Chang Gung Memorial Hospital-Kaohsiung Medical Center, Chang Gung University College of Medicine, Kaohsiung, 83301 Taiwan; Department of Child Psychiatry, Chang Gung Memorial Hospital, Chang Gung University College of Medicine, Taoyuan, 33302 Taiwan

**Keywords:** *PARK2*, Autism spectrum disorder (ASD), Copy number variations (CNVs), Family study, Gene expression

## Abstract

**Background:**

Autism spectrum disorder (ASD) is an early-onset neurodevelopmental disorder with complex genetic underpinning in its etiology. Copy number variations (CNVs) as one of the genetic factors associated with ASD have been addressed in recent genome-wide association studies (GWAS). However, the significance of CNV has not been well investigated in non-Caucasian ASD population.

**Methods:**

To identify the pathogenic CNVs responsible for ASD in Han Chinese, we performed a segment-based GWAS of CNV in 335 ASD cases and 1093 healthy controls using Affymetrix single nucleotide polymorphism (SNP) array by focusing on case-specific CNVs. *PARK2* was one of the important genes with several case-specific regions overlapped on it. The findings were validated in the initial screen sample set and replicated in another sample set by real-time quantitative PCR (qPCR).

**Results:**

A total of six CNVs at 6q26 that spanned different exons of *PARK2* were identified. The *PARK2* expression level was down-regulated at exon-dependent manner in cases with either deletion or duplication. The result revealed that the gene function might be disrupted by exonic deletion and duplication. We also observed that the ASD case with exonic duplication demonstrated a more severe interference of *PARK2* expression and the clinical feature than the ones with deletion at the exons 2–4 of the *PARK2* gene.

**Conclusions:**

Our finding provides evidence to support that CNVs affecting *PARK2* function might contribute to genetic etiology of a proportion of cases with ASD. The intriguing results of this work warrant further study on characterizing the functional impact of various exonic CNVs on the *PARK2* gene.

**Trial registration:**

ClinicalTrials.gov NCT00494754

**Electronic supplementary material:**

The online version of this article (doi:10.1186/s13229-016-0087-7) contains supplementary material, which is available to authorized users.

## Background

Autism spectrum disorder (ASD) is a common, early-onset, long-term impairing neurodevelopmental disorder with complex genetic underpinning in its etiology [1, 2]. Recent massive improvement in cytogenetic analysis enhanced the resolution and accuracy in detecting genomic structural changes. Copy number variations (CNVs) are the submicroscopic chromosomal deletions or duplications that affect more than 1000 base pairs of deoxyribonucleic acid (DNA) sequence and alter normal gene expression either by removing or adding the copies of a gene or multiple genes [3, 4]. Accumulated evidence from genome-wide CNV studies showed that ASD patients carried higher CNV burden than healthy controls [5], and up to 10–20 % of ASD cases carried one or more CNVs that contribute to the phenotypes [6].

Evidence from several studies of genome-wide analyses of CNVs supports that rare CNVs, either de novo or inherited, are associated with ASD [7–9]. Sebat et al.’s report of rare de novo CNVs in ASD using array comparative genomic hybridization (aCGH) method [7] was supported by a large-scale CNV study implemented by the Autism Genome Project (AGP) showing that the difference was mainly contributed by the deletion-type CNVs [8]. Sander et al. reported that duplications of 7q11.23, the region of Williams Syndrome, were strongly associated with ASD and that large de novo CNVs encompassing multiple genes were more pathogenic in ASD (OR = 5.6, CI = 2.6–12.0, *P* = 2.4 × 10^−7^) [9]. Many of these loci/genes are relevant to the process of neurotransmission [10–12], synapse formation [10, 11], or protein ubiquitination [12]. Functional levels of genes change with CNV (termed *dosage* sensors) may additionally contribute to the etiology of ASD [10–12].

The *PARK2* gene (OMIM*602544) is one of neurodevelopmental genes that was originally discovered as one of the causes of early-onset Parkinson disease (PD) [13] and was subsequently reported to be associated with schizophrenia [14], ASD [15], and attention-deficit/hyperactivity disorder (ADHD) [16]. Glessner et al. (2009) identified deletions in the *PARK2* gene in seven ASD patients, but none in controls (*P* = 0.0047) [15]. Scheuerle et al. then found two ASD patients with deletion and duplication of *PARK2* [17], and suggested that distinct phenotype in patients might be due to different CNV types in *PARK2* and the duplications may be equally causative for ASD [17]. Overall, these two studies provide some evidences to support *PARK2* as a pathogenic gene of ASD [15, 17].

The majority of population-based studies of genome-wide CNVs are about European ancestry but there is no such study in the Asian population. Therefore, it is of great importance to discover candidate CNVs that are responsible for ASD in Han Chinese. With the identification of pathogenic CNVs, clinical diagnosis, and treatment for ASD should be largely improved. Here, we report the study using genome-wide association analysis of CNVs in 335 ASD cases and 1093 healthy controls of Han Chinese ancestry in Taiwan. We identified a marginally significant (not adjusted for multiple testing) ASD-associated gene, *PARK2*, which was co-localized with a CNV region specific to ASD. The finding was further replicated in an independent sample set of 301 ASD cases and 301 healthy controls. We also investigated how the expression of the *PARK2* gene was affected by the intragenic CNVs covering various exons. Finally, we linked the clinical features of ASD to the *PARK2* CNVs and proposed the possible pathogenic effect of various exonic CNVs of *PARK2*.

## Methods

### Participants

#### ASD cohort

A total of 335 Han Chinese patients with ASD, aged 9.39 ± 4.04 years (male 89.3 %) were recruited in Taiwan for the genome-wide CNV comparison. They were clinically diagnosed with autistic disorder according to the DSM-IV and confirmed by using the Chinese version of the Autism Diagnostic Interview-Revised (ADI-R) [18]. The ADI-R interviews revealed the 335 probands scored 20.43 ± 6.12 in the “qualitative abnormalities in reciprocal social interaction” (cut-off = 10), 14.75 ± 4.32 in the “qualitative abnormalities in communication, verbal” (cut-off = 8), and 6.95 ± 2.47 in the “restricted, repetitive and stereotyped patterns of behaviors” (cut-off = 3) currently if ages of 5 or younger or at their age of 4 to 5 years old with the typical symptoms if older than 5 years old (Table 2). They were recruited from Department of Psychiatry of National Taiwan University Hospital (NTUH), Chang Gung Memorial Hospital (CGMH), Taoyuan, and Taoyuan Mental Hospital (TMH), Taiwan. The patients diagnosed as fragile X, Rett’s disorder, or other known chromosome/genetic disorders were excluded from the study [19]. Their parents also reported about the patients’ autistic behaviors on the Social Responsiveness Scale (SRS) [18], and the cognitive functions assessed by the Weschler Intelligence Scale for Children-3^rd^ edition (WISC-III) and Wisconsin Card Sorting Test (WCST). The detailed description of the current and past ADI-R, SRS, WISC-III, and WCST is provided in Table 2.

#### Healthy control cohort

One thousand ninety-three individuals (aged 68.07 ± 10.12 years, male 48.0 %) from the Han Chinese Cell and Genome Bank (HCCGB) in Taiwan served as controls in the CNV screening stage [20]. This control cohort received physical check-up and questionnaire screening to confirm that they did not have any physical condition.

#### The sample for replication

The ASD cohort for replication consisted of 301 independent patients (aged 11.25 ± 5.47 years, male 88.4 %) recruited from the same places as the initial ASD cohort and received the same assessments. The ADI-R interviews revealed that the 301 probands for replication scored 20.77 ± 6.28 in the “qualitative abnormalities in reciprocal social interaction” (cut-off = 10), 15.87 ± 4.63 in the “qualitative abnormalities in communication, verbal” (cut-off = 8), and 7.29 ± 2.65 in the “restricted, repetitive and stereotyped patterns of behaviors” (cut-off = 3) currently if ages of 5 or younger or at their age of 4 to 5 years old with the typical symptoms if older than 5 years old (Table 2). The healthy controls for replication included 301 unrelated individuals (controls aged 19.59 ± 8.90 years, male 64.1 %) recruited at NTUH, Taipei, Taiwan, either by teachers’ referral or advertisement. Their lifetime and current mental status were clinically evaluated by the corresponding author and confirmed by psychiatric interviews. Their family history of mental disorders was also screened by clinical interviews.

The study protocol was approved by the Research Ethics Committee at each participating hospital and Academia Sinica (National Taiwan University Hospital, 9561709027; Chang Gung Memorial Hospital-Linkou, 93-6244; Taoyaun Psychiatric Center, Ministry of Health and Welfare, C20060905; Kaohsiung Chang Gung Memorial Hospital, 99-1548A3; Academia Sinica, AS-IBMS-MREC-91-10). Written informed consent was obtained from the parents and participants after the purposes and procedures of the study were explained, and the voluntary participation was assured.

### Genome-wide SNP genotyping and data analysis

Genomic DNA was extracted from the peripheral blood of the ASD subjects and from EBV-transformed B cells of the healthy controls by PUREGENE Genomic DNA Purification Kit (Qiagen, Valencia, CA). The DNA samples were subjected to genotyping using Affymetrix Genome-Wide Human SNP Array 6.0 (Affymetrix, Santa Clara, CA) according to the manufacturer’s instructions and performed in the National Center for Genome Medicine at Academia Sinica, Taipei, Taiwan (http://ncgm.sinica.edu.tw/ncgm_02/index.html). The hybridization intensities were captured by GeneChip Scanner 3000 (Affymetrix, Santa Clara, CA). CNVs were called using Affymetrix Genotyping Console software v.4.1 (Affymetrix, Santa Clara, CA). Genes overlapped with the CNV regions were reported according to UCSC genes (NCBI37/hg19). Details on genotyping and sample quality control, the procedures for CNV detection and extraction, and the global CNV burden measurement are provided in Additional file 1: Figure S1 and Additional file 1: Table S1.

### Case-specific CNV locus

The segment-based association analysis was conducted using Python (https://www.python.org). CNV regions on autosomes were analyzed in all samples while CNV regions on sex chromosomes were analyzed in male samples only. Duplication-type CNV and deletion-type CNV were treated as different CNVs. CNV regions identified in each individual were stacked and partitioned at each unique start and stop site for the CNV regions to form non-overlapping genomic segments for frequency calculation. A total of 38,660 individual autosomal CNV regions formed 7883 CNV segments, and a total of 1999 individual sex-chromosome CNV regions identified in male samples formed 615 CNV segments. CNV segments that were detected only in cases were defined as case-specific CNVs. The case-specific CNV segments that were flanking to each other or belonged to the same gene were manually merged into case-specific CNV loci for report.

### Validation and replication analysis of CNVs

The case-specific CNVs were validated by SYBR-Green based genomic quantitative PCR (qPCR) using ABI StepOne Plus system (Applied Biosystems, Forster, CA). Primer pairs specific to various exons of the *PARK2* gene (NM_004562), the *FARP2* locus on chromosome 2 and the *GAPDH* locus on chromosome 12 were used as internal controls for qPCR. A total of 10 ng of genomic DNA was used in each PCR reaction, which was conducted in triplicate for both target locus and internal control loci. The fold change of copy number was calculated by comparative Ct method. The relative fold change to normal subject was determined as 2^−∆∆Ct^. Samples with fold change greater than 1.25 (duplication) or less than 0.75 (deletion) are considered to have a copy number change.

At replication stage three additional pairs of primers were designed to cover exons 1, 2, and 4 of the *PARK2* gene, respectively. The information of these primers is listed in Additional file 1: Table S2.

### Gene expression analysis

Total ribonucleic acid (RNA) was extracted from peripheral blood cells by RNeasy Miniprep Kit (Qiagen system) following the manufacturer’s instructions. Of purified RNA sample, 1.2 μg was used for complementary DNA (cDNA) synthesis by using High-Capacity cDNA Reverse Transcription Kit (Applied Biosystems). The cDNA product was subjected to SYBR-Green based qPCR by ABI StepOne Plus system (Applied Biosystems) for *PARK2* expression analysis. *GAPDH* was used as an internal control. A 20-fold dilution of cDNA product was used in each PCR reaction in triplicate for both target gene and internal control gene. The information of the primers is listed in Additional file 1: Table S2. The probands, *PARK2* CNV carriers in the families, non-carriers in the families, and four unrelated controls without *PARK2* CNV were assayed individually. Relative fold change of *PARK2* exon expression was calculated by 2^−∆∆Ct^ method using individuals without *PARK2* CNV as expression baseline.

### Statistical analysis

Two-tailed Fisher’s exact test was performed to evaluate the associations of individual CNV segments and case-specific CNV loci with ASD. Student *t* test was used to compare the messenger ribonucleic acid (mRNA) expression level between the two groups.

## Results and discussion

### Case-specific CNV loci

The present study focused on the case-specific CNV segments, which were detected in at least one case but none in healthy controls from the screening stage (Additional file 1: Figure S2). The flanking case-specific CNV segments with the same type of CNV were merged into CNV loci for statistical analysis and annotation. A total of 461 case-specific CNV loci overlapped with genes were identified (Additional file 1: Table S3). These case-specific CNV loci consisted of 196 deletions (42.5 %) and 265 duplications (57.5 %), and most of them were individually rare (<1 % of cases). These CNV loci spanned 0.3 to 4623.3 kb (average CNV locus size: 143 kb) and overlapped with exons of a single gene to more than 40 genes.

Among the identified case-specific CNV loci, 17 were located in six chromosomal regions of the well-known ASD-associated CNV, 1q21.1, 15q11.2-13.1, 15q13.3, 16p11.2, 22q11.21, and 22q13.33 (Additional file 1: Table S4). Our findings that there were case-specific CNVs located in these 17 ASD-associated loci replicate those findings of pathogenic CNV loci from previous studies [5, 7–9, 15, 21–24]. In addition to the known CNV loci associated with ASD, we identified 17 marginally significant (not adjusted for multiple testing) case-specific CNV loci at which duplication and deletion were detected in at least four cases (patient frequency (*F*_patient_) > 1 %; *P* < 0.01) (Table 1). Genes overlapped with these case-specific CNV loci could be candidate pathogenic genes for ASD (Table 1). Among them, the *PARK2* gene is the only one that has been reported to be associated with ASD.

**Table 1:** Selected case-specific copy number variation loci

Locus	Position (start–end)	Gene	Gain_case	Loss_case	Gain_control	Loss_control
1p36.21	13377035–13538299	*PRAMEF8,PRAMEF9,PRAMEF13,PRAMEF19,PRAMEF16,PRAMEF20*	6	0	0	0
1p36.13	17262235–17295200	*CROCC*	4	0	0	0
1q25.1	174802140–174802829	*RABGAP1L*	0	4	0	0
2p11.2-p11.1	91801473–92136779	*LOC654342,Mir_544,GGT8P,ACTR3BP2*	7	0	0	0
3p12.3	75639495–75930920	*MIR1324,FLJ20518,LOC401074,ZNF717,MIR4273*	10	0	0	0
3q22.1	129806912–129914958	*ALG1L2, FAM86HP*	27	1	0	0
4p16.1	9486075–9744591	*MIR548I2,AB059369*	8	1	0	0
4p16.3	3885796–4190560	*DQ584669,FAM86EP,BC042823,OTOP1*	14	0	0	0
4q13.2	69371991–69410211	*UGT2B15, UGT2B17*	0	5	0	0
6q26	162187125–162680305	*PARK2*	1	3	2	0
8p23.1	12542721–12587390	*LONRF1,MIR3926-1,MIR3926-2*	5	0	0	0
9q13	68176154–68683835	*AK308561,BC080605,LOC642236*	17	0	0	0
12p13.31	8378239–8536600	*LINC00937, FAM86FP, FAM90A1*	5	0	0	0
14q11.2	24429298–24513905	*DHRS4,DHRS4L2,DHRS4L1*	1	11	5	10
19q13.42	55360538–56717229	*>60 genes*	4	0	0	0
21q11.2	14456404–14594223	*ANKRD30BP2*	8	1	0	0
22q11.23	24396802–24404830	*GSTTP2*	0	4	0	0

### Validation and replication analysis of CNVs at the *PARK2* locus

**Fig. 1:**
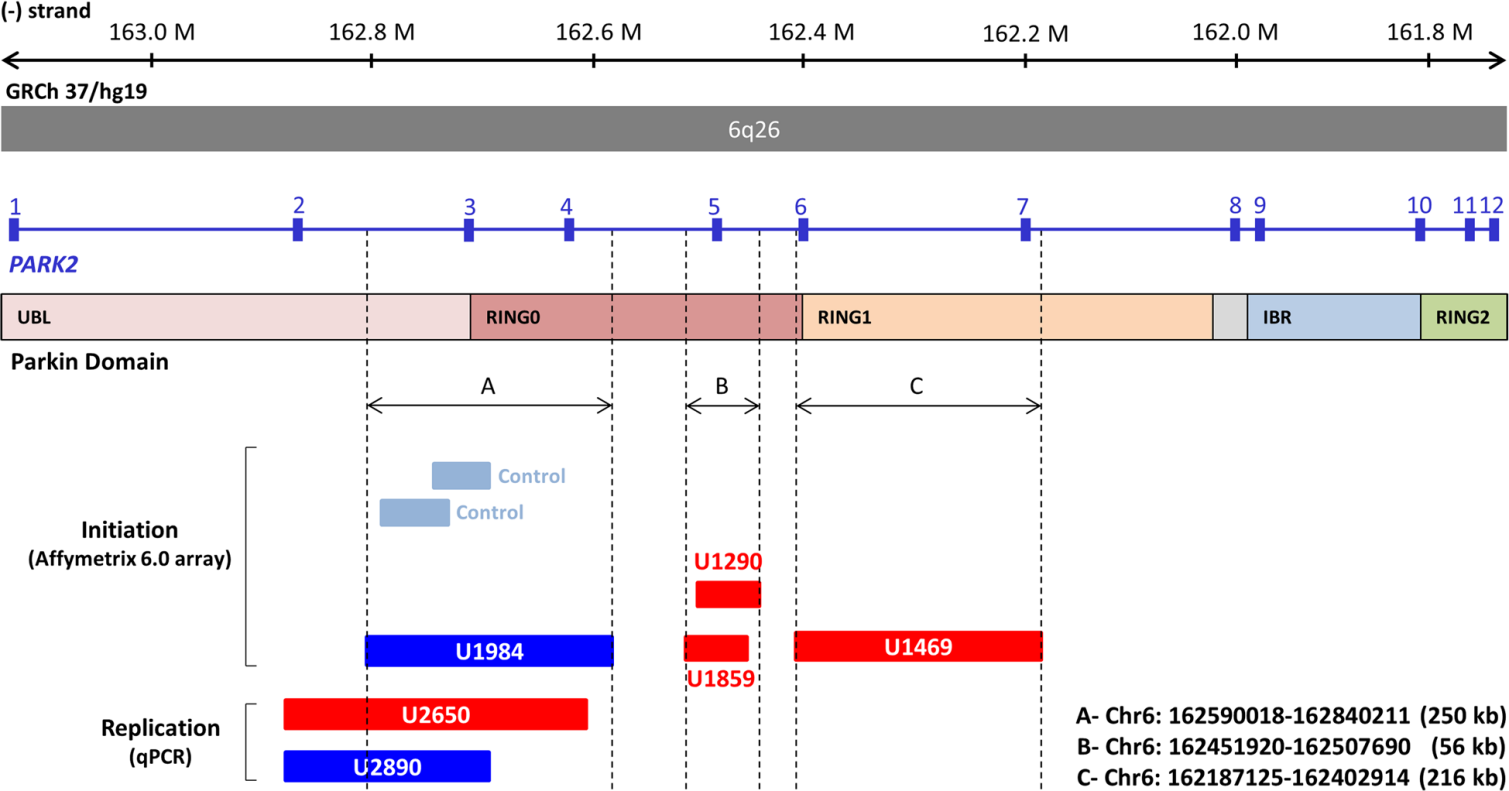
Genomic location of CNVs at the *PARK2* locus. *Red bars* and *dark blue bars* represent deletions and duplication, respectively, at the *PARK2* gene detected in the cases. *Light blue bars* represent duplication identified in healthy controls. Transcript and genomic coordinates corresponds to human genome Build 37 (hg 19). The A region overlaps with exons 3–4, which corresponds to the UBL and RING0 domain of the Parkin protein. The B region overlaps exon 5, which corresponds to the RING0 domain of the Parkin protein. The C region overlaps with exons 6–7, which corresponds to the RING0 and RING1 domain of the Parkin protein

**Fig. 2:**
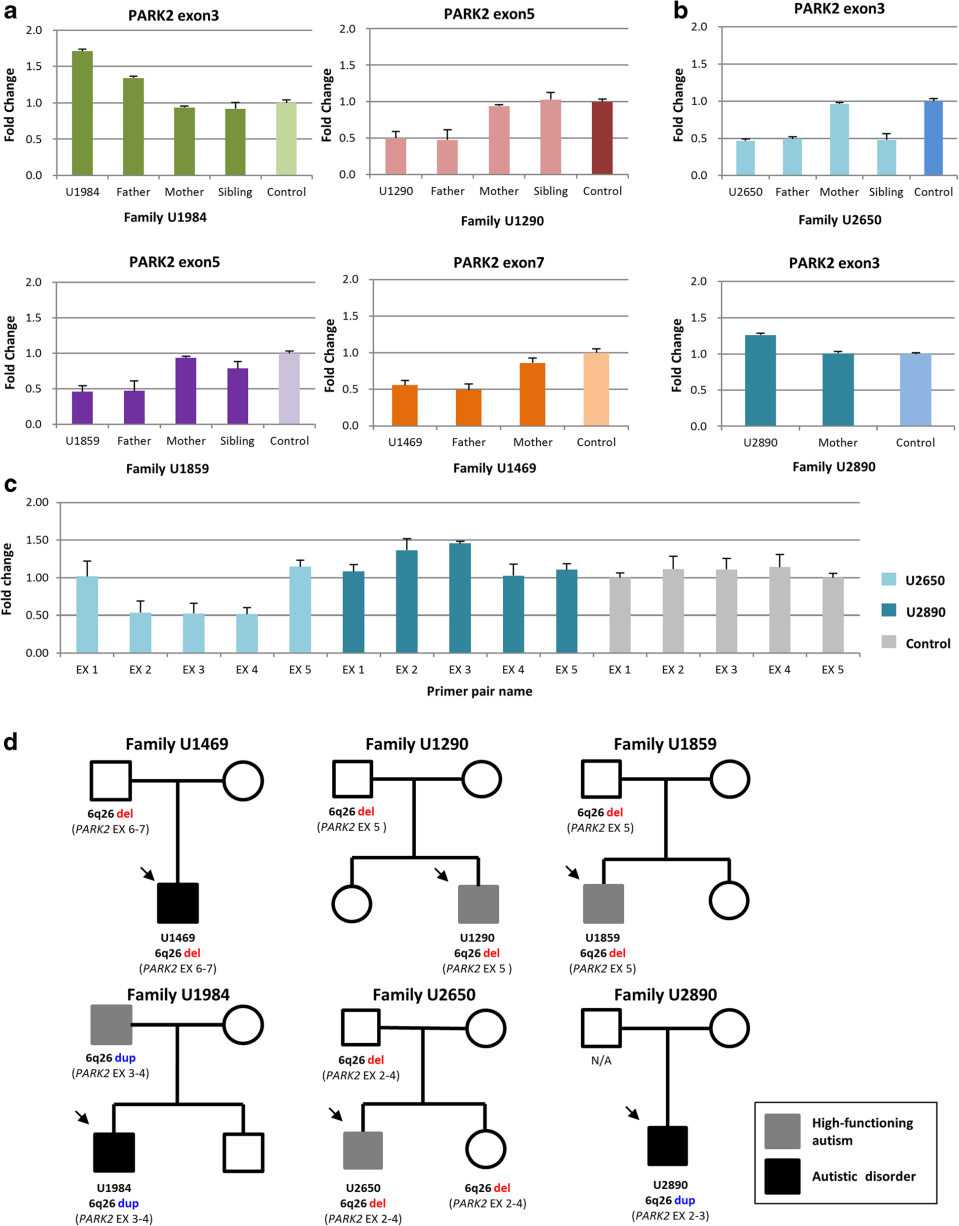
Detection of CNVs at the *PARK2* locus by genomic qPCR. **a** SYBR-based qPCR assays were performed to validate exonic CNVs at the *PARK2* locus in the cases and the family members. Data is presented as mean ± SD. **b** Additional two ASD cases with CNV at exon 3 of the *PARK2* locus were detected. **c** Copy number changes of five exons (exons 1 to 5) of the *PARK2* gene were measured to determine the spanning of the CNV region in cases U2650 and U2890. *EX* exon. Data is presented as mean ± SD. **d** Pedigrees of probands with *PARK2* exonic CNVs. *Arrows* indicate the proband in each family. *N/A* genomic DNA was not available, *del* deletion, *dup* duplication. Exons involved are determined by genomic qPCR

A total of four exonic CNVs within the *PARK2* locus were identified in ASD cohort at the initial screening stage (three deletions, one duplication, *F*_patient_ = 1.19 %). Each of them overlapped with different parts of the *PARK2* gene and was categorized into three coverage regions: A, B, and C (Fig. 1). With the three regions as baits, two duplications within the A region were identified in two healthy controls (*F*_control_ = 0.18 %) (Fig. 1). Nevertheless, both of them were shorter, and only one overlapped with the exon 3 of *PARK2*. The prevalence of CNVs at the entire *PARK2* locus remained significant in ASD cases compared to the controls (*P* = 0.030). Copy number status at the *PARK2* locus in the initial samples was validated by genomic qPCR (Fig. 2a and Additional file 1: Figures S3 and S4).

CNV detection in the replication cohort focused on exons in regions A, B, and C by using genomic qPCR with the same primer pairs used for validation. We detected two exonic CNVs (one deletion, one duplication, *F*_patient_ = 0.66 %) at the A region carried by two ASD cases (Fig. 2b) while no exonic CNVs were detected in controls. The probability that the CNVs identified in the two cases might extend to adjacent exons was tested by using genomic qPCR with primer pairs specific to exons 1, 2, 4, and 5, respectively. The results showed that U2650 indeed had a deletion spanning exons 2–4 (Figs. 1 and 2c). The *PARK2* transcript missing exons 2–4 is predicted to result in frameshift and lead to a nonsense mutation at codon 9 in the mutant mRNA. U2890 had a duplication overlapped with exons 2–3 (Figs. 1 and 2c). Taken together, the frequency of exonic CNVs at the *PARK2* locus was significantly greater in ASD cases (6/636) than in controls (2/1394, *P* = 0.014, Additional file 1: Table S5). Of note, five probands revealed that the *PARK2* CNVs were paternally inherited (Fig. 2a, b, d) except that the DNA sample was not available from the father of U2890. We could only confirm that the duplication was not inherited from the mother (Fig. 2b). In addition, we observed that the sister of U2650, who had the same *PARK2* exonic deletion, did not demonstrate any autistic feature. In contrast, the father of U1984 (duplication) was clinically diagnosed with high-functioning autism (Fig. 2a, d).

### *PARK2* expression analysis

**Fig. 3:**
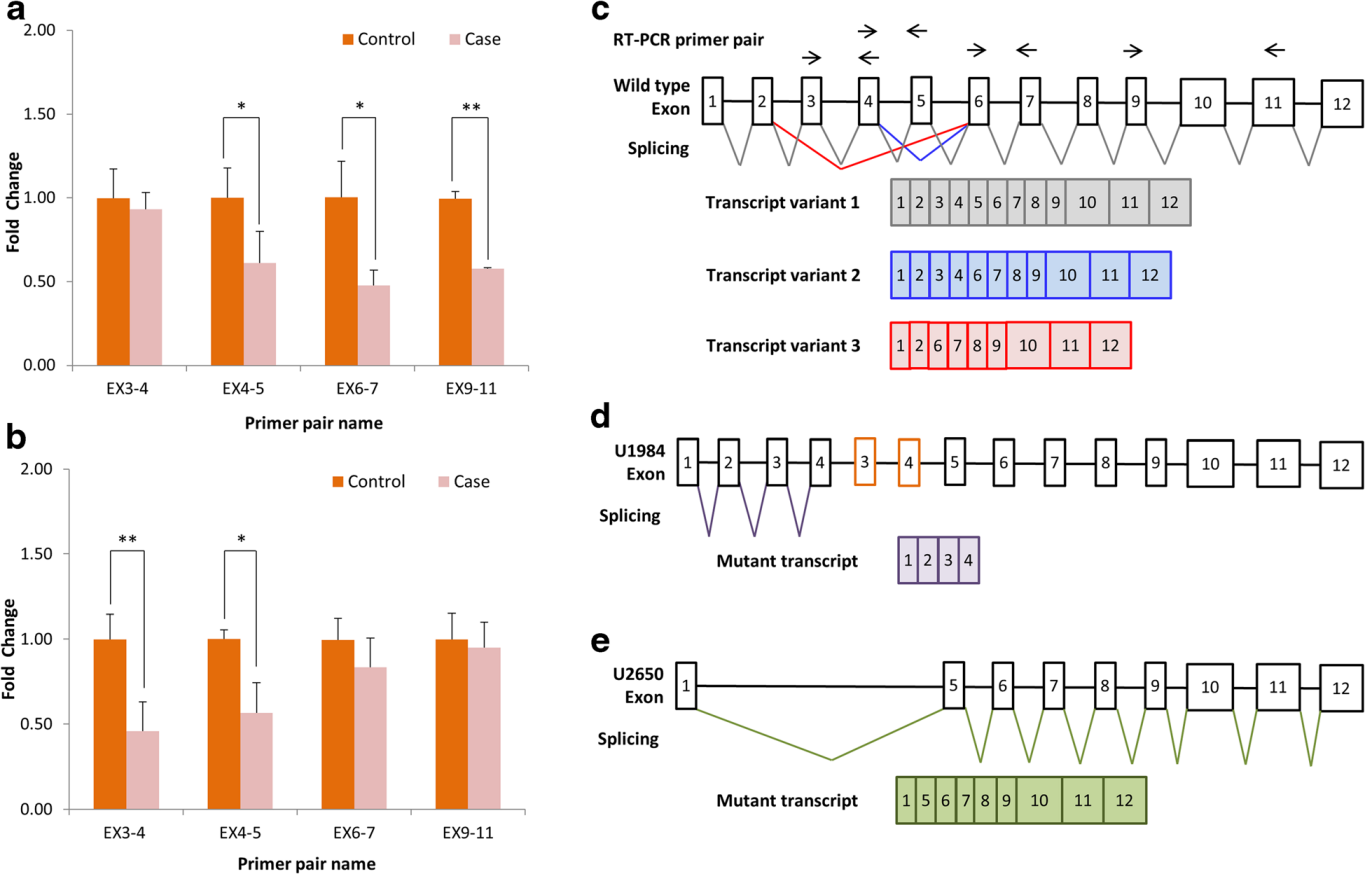
Expression analysis of *PARK2*. The expression level of *PARK2* transcript was detected using four primer pairs specific to EX3-4, EX4-5, EX6-7, and EX9-11, respectively. Decreased expression level of specific *PARK2* exons was observed in (**a**) case group of Family U1984 and (**b**) case group of Family U2650 (*P < 0.05, **P < 0.01). **c** Alternative splicing of *PARK2* gene produces three known transcript variants (transcript variant 1: *grey*, transcript variant 2: *blue*, transcript variant 3: *red*) according to RefSeq Gene. *Arrows* on the top of the gene locus indicate the primer pairs used for expression detection. **d** The proposed genomic DNA rearrangement and *PARK2* mutant transcript (*purple*) in the proband U1984 with duplication of EX3-4 on *PARK2* based on the result of qRT-PCR. The frame was drawn in *orange* to represent the EX3-4 duplication. **e** The proposed *PARK2* mutant transcript (*green*) in the proband U2650 with deletion of EX2-4 on *PARK2* based on the result of qRT-PCR

To assess how this exonic CNV affected gene function, we performed quantitative RT-PCR (qRT-PCR) to compare the mRNA expression level of *PARK2* in individuals with and without exonic CNV. Family U1984 and Family U2650 consented to this experiment and were found to possess two different types of CNVs covering exon 3 and exon 4 which belonged to the A region at the *PARK2* locus. We used two primer pairs specific to the CNV-affected regions (exons 3–4 and exons 4–5), and two primer pairs specific to the CNV-unaffected regions (exons 6–7 and exons 9–11) for the quantification of *PARK2* mRNA expression, respectively. We observed a fold change of 0.61 for exons 4–5, 0.48 for exons 6–7, and 0.58 for exons 9–11 in the proband and the father of Family U1984 when compared with unaffected controls (*P* = 0.014, *P* = 0.026, and *P* = 0.01, respectively). In contrast, no difference in the expression level of exons 3–4 was detected (Fig. 3a).

We observed a fold change of 0.46 for exons 3–4 and 0.56 for exons 4–5 in the proband and deletion-carriers of Family U2650 when compared with those of unaffected controls (*P* = 0.014 and *P* = 0.004, respectively). However, no significant difference of the expression level of exons 6–7 or exons 9–11 between controls and deletion-carriers was found (Fig. 3b).

There were three transcript variants of the *PARK2* gene according to the RefSeq Gene database (Fig. 3c). The exon-dependent change of *PARK2* expression level in the probands and carriers of Family U1984 and Family U2650 implies that both the wild-type and mutant genomic copy of the *PARK2* gene were expressed. Based on the qRT-PCR result, it is likely that the transcript variant 1 from the wild-type genomic copy (Fig. 3c) and mutant transcript consisting of exons 1–4 resulted from the mutant genomic copy (Fig. 3d) were expressed in the proband and the father of Family U1984. Likewise, the transcript variant 1 from the wild-type genomic copy (Fig. 3c) and mutant transcript missing exons 2–4 resulted from the mutant genomic copy (Fig. 3e) were suspected to be expressed in the individuals of Family U2650 with deletion of exons 2–4.

### Clinical features of patients with *PARK2* exonic CNVs

Pedigrees of these six families with male probands are illustrated in Fig. 2d. The age at recruitment (6–21 years old), clinical diagnosis, intelligence quotient (IQ) profiles (full-scale IQ, 83-117), subscores of the ADI-R and SRS, and WCST indexes of the five probands (except U2890) are summarized in Table 2. Based on the clinical assessment, all the parents of the six patients did not have ASD except that the father of U1984 had high-function autism (HFA) and the father of U1469 had autistic trait assessed by the autism-spectrum quotient (AQ) (Additional file 1: Table S6).

**Table 2:** Clinical features of male probands with PARK2 exonic CNV

	U1469	U1290	U1859	U1984	U2650	U2890	Initial sample 335 probands (Mean ± SD)	Replication sample 301 probands (Mean ± SD)
Age (year) of diagnosis	6	2	8	7	8	–	–	–
Age (year) of recruitment	6	15	10	7	10	21	9.39 ± 4.04	10.66 ± 5.36
Clinical diagnosis	Autism	HFA	HFA	Autism	HFA	Autism	–	–
CNV type/region	Loss/exons 6–7 (C region)	Loss/exon 5 (B region)	Loss/exon 5 (B region)	Gain/exons 3–4 (A region)	Loss/exons 2–4 (A region)	Gain/exons 2–3 (A region)		
Inheritance	Paternal	Paternal	Paternal	Paternal	Paternal	Unknown		
Speech delay	N	Y	Y	Y	N	–		
Other	–	–	–	Epilepsy	–	–		
Autism Diagnostic Interview-Revised (ADI-R) (current/past)								
Qualitative abnormalities in reciprocal social interaction (cut-off = 10)	19/26	11/21	10/19	13/27	5/4	–	12.39 ± 5.76/20.43 ± 6.12	12.51 ± 5.69/20.77 ± 6.28
Qualitative abnormalities in communication, verbal (cut-off = 8)	10/13	5/12	8/14	13/16	9/13	–	11.48 ± 3.83/14.75 ± 4.32	13.10 ± 4.59/15.87 ± 4.63
Restricted, repetitive, stereotyped patterns of behaviors (cut-off = 3)	7/7	2/5	3/5	6/10	4/6	–	5.43 ± 2.48/6.95 ± 2.47	5.61 ± 2.46/7.29 ± 2.65
Abnormality of development evident at or before 36 months (cut-off = 1)	2	5	2	4	1	–	3.44 ± 1.53	3.18 ± 1.63
Social Responsiveness Scale (SRS) Raw (*t* score)								
Social communication (score 0–84)	42 (84.81)	42 (89.91)	34 (74.08)	49 (92.51)	17 (56.50)	–	37.28 ± 14.13	41.04 ± 15.07
Stereotyped behaviors/interest (score 0–39)	24 (83.74)	24 (90.13)	17 (70.30)	27 (89.47)	10 (57.17)	–	18.88 ± 7.00	20.04 ± 7.42
Social awareness (score 0–33)	24 (65.07)	28 (69.01)	23 (62.20)	21 (60.23)	16 (51.36)	–	21.70 ± 4.97	21.85 ± 5.45
Social emotion (score 0–24)	16 (71.43)	15 (72.56)	15 (69.22)	14 (66.97)	7 (51.44)	–	11.45 ± 4.55	12.64 ± 4.76
Total score (score 0–180)	106 (83.32)	109 (88.77)	89 (72.90)	111 (85.76)	50 (55.25)	–	89.24 ± 25.84	95.57 ± 28.56
Intelligence quotient (IQ)								
Verbal IQ (range)	117	122	107	84	121	–	95.08 ± 23.79 (44–148)	94.80 ± 23.79 (40–145)
Performance IQ (range)	90	94	95	85	111	–	96.74 ± 21.04 (41–145)	94.96 ± 21.28 (40–139)
Full–scale IQ (range)	105	109	102	83	117	–	94.85 ± 22.55 (40–148)	93.56 ± 23.14 (40–141)
Wisconsin Card Sorting Test (WCST) (%)								
Total errors	63 (4 %)	35 (21 %)	19 (84 %)	48 (14 %)	16 (92 %)	–	44.11 ± 26.61	39.75 ± 25.25
Perseverative response	34 (14 %)	20 (21 %)	6 (96 %)	55 (2 %)	8 (88 %)	–	27.32 ± 25.29	25.95 ± 26.15
Perseverative errors	30 (13 %)	19 (18 %)	6 (97 %)	43 (3 %)	8 (87 %)	–	23.51 ± 19.31	22.23 ± 19.95
Nonperseverative errors	33 (4 %)	15 (32 %)	13 (53 %)	5 (92 %)	8 (82 %)	–	20.60 ± 15.71	17.60 ± 12.07
Conceptual level responses	18 (4 %)	53 (18 %)	77 (82 %)	39 (16 %)	81 (91 %)	–	58.44 ± 23.96	59.52 ± 21.03
Categories completed	2 (>16 %)	6 (>16 %)	6 (>16 %)	1 (>16 %)	6 (>16 %)	–	4.12 ± 2.03	4.39 ± 2.02

*CNV* copy number variation, *ADI-R* the ADI-R interviews revealed the 636 patients scored 20.7 ± 6.5 in the “qualitative abnormalities in reciprocal social interaction” (cut-off = 10), 15.2 ± 4.7 in the “qualitative abnormalities in communication, verbal” (cut-off = 8), and 7.0 ± 2.7 in the “restricted, repetitive, and stereotyped patterns of behaviors” (cut-off = 3), *SRS* the Chinese version of the Social Responsiveness Scale (60 items; score 0 (never true) - 3 (almost always true); total scores can range from 0 to 180) and the *t* score was generated by comparing to Taiwanese norm, *HFA* high function autism. *WCST* percentile ≤ 10 %: poor performance, percentile 11~90 %: normal range, percentile ≥ 91 %: great performance

Both U1984 and U2650 were found to have CNVs at the A region of *PARK2* with different exonic CNV patterns (duplication/deletion). U1984 was born in a family with unrelated parents and had an unaffected typically developing younger brother. He presented speech delay (two words and simple sentence at age 4), stereotyped and socially inappropriate speech, abnormal social reciprocity, and restricted/repetitive behaviors and interests. He demonstrated severe autistic symptoms compared to U2650 in a wide range of autistic features at his age of 4–5 years old and current status (Table 2). He suffered from the first attack of absence seizure at his age of 2.5 years old but no anti-epileptic drug was used. U1984 had a relatively lower IQ profile (Full-IQ = 83) than the other four probands, and his performance of WCST showed increased perseverative response and errors, suggesting an impaired cognitive flexibility.

U2650 (deletion in *PARK2*), diagnosed as HFA, showed no speech delay and less severe autistic symptoms assessed by the ADI-R and SRS compared to the other probands. His key features were socially inappropriate communication and interactions, a lack of non-verbal communication (e.g., eye contact, gesture, or distal index pointing), and stereotyped pattern of interests, verbal rituals, and schedules (Table 2). He demonstrated above average IQ profiles (Full-IQ = 117) and did not have impaired WCST performance. Although his sister carries the same deletion in *PARK2*, the clinical assessment and parental reports revealed her as a typically developing child without any autistic symptom.

U1469, who carried an exonic deletion spanning exons 6–7 of *PARK2*, showed abnormal social reciprocity and communication, neither sharing nor comforting, sameness in a wide range of behaviors, and unusual sensory interests, which had been clearly noted before 3 years old. Despite IQ profiles within the normal range, he demonstrated impaired cognitive flexibility as assessed by the WCST (Table 2). His *PARK2* deletion was inherited from his father, who showed some autistic trait such as restricted interests, social inflexibility, and concrete thought based on clinical assessment.

Both U1290 and U1859, diagnosed with HFA, had exonic deletions spanning exon 5 of *PARK2* (Table 2). U1290 exhibited speech delay and started to combine two words to make simple sentences until age 4; U1859 was observed to have speech delay. They both had difficulty in offering comfort to other people, engaging reciprocal social interactions, developing peer relationships, and imitating behaviors/action. The cognitive assessments of these two probands did not reveal any abnormal results, but U1859 revealed better WCST performance than U1290.

In this study, we focused on the case-specific CNVs rather than rare CNVs (<1 % frequency in total samples). With this approach, we have sufficient power to catch variants that exist only in ASD cases without losing any important CNVs that have >1 % frequency. For example, we found that the CNVs on chromosome 3q22.1 and 9q13 were presented at more than 1 % of frequency in the total samples, but indeed were contributed only from the cases (Table 1). Among the candidate genes overlapped with case-specific CNV loci, we focused on *PARK2*, which has been known to be associated with ASD [15], other neuropsychiatric disorders [13, 14, 16], and neuronal functions in Caucasian population [25–28]. With two independent sets of case-control cohorts, we demonstrated that the frequency of CNVs affecting *PARK2* gene was significantly higher in individuals with ASD compared to controls (0.94 vs. 0.14 %, *P* = 0.014). Our findings strongly suggest that CNVs at the *PARK2* locus contribute to the susceptibility of ASD in the Han Chinese population residing in Taiwan. Of note, in the present study, there were 12 people who carried *PARK2* CNVs from six families, and seven of them were diagnosed with ASD, and the *PARK2* CNVs were often transmitted by unaffected parents. The study of Icelandic population by Huttenlocher and her colleagues demonstrated that the frequency of heterozygous *PARK2* CNVs was significantly higher in patients with Parkinson’s disease (PD) compared to controls (OR = 1.69, *P* = 0.03) [29]. They concluded that heterozygous carriers for exonic CNVs in *PARK2* were at an increased risk of developing PD. On the other hand, their results also clearly demonstrated that there was a significant proportion of individuals who carried heterozygous *PARK2* CNV who did not develop PD after age 65. Therefore, based on their results, the penetrance for PD from heterozygous CNVs in *PARK2* would not be high. In this report, the six indexed ASD cases, one affected father, and the five unaffected carriers were all heterozygote of *PARK2* CNVs. Therefore, we speculate that the penetrance for ASD from heterozygous *PARK2* CNV was not high, either.

Our result that *PARK2* is strongly suspected as a candidate gene for ASD is in line with Glessner’s observation [15]. However, the enriched CNVs in their study were located at the intronic region and might not directly influence the expression or function of the gene [15]. Moreover, a comprehensive study of *PARK2* showed that CNVs involving *PARK2* were identified in 1 % of control subjects, and CNVs involving exons 2–4 were well-tolerated while exons 5–12 were not. Kay and colleagues suggested that the mutations affecting exons 5–12, the coding region for highly conserved function domains of Parkin, might be more deleterious [30]. This suggestion may explain our findings on the clinical features of four probands: U1469 (exons 6–7) had more severe autistic symptoms than U1290 and U1859 (exon 5), and U2650 (exons 2–4) had less severe autistic symptoms than other probands.

The *PARK2* transcript missing exons 2–4 is predicted to result in a frameshift and lead to a nonsense mutation at codon 9 in the mutant mRNA. Similar to the prediction, we observed that the mRNA expression levels in U2650 and carriers with EX2-4 deletion were changed in an exon-dependent manner: decreased by half in the CNV-affected exons but was not affected in the CNV-unaffected exons, suggesting that a mutant *PARK2* transcript without EX2-4 is generated (Fig. 3e). However, surprisingly, nonsense-mediated decay did not occur with this transcript. We speculate that the mutant transcript would be translated into a non-functional protein, and the individual carrying this mutation has half dose of functional *PARK2* proteins. However, U2650 was one of the less severe autistic symptoms of patients with *PARK2* exonic CNV in this study, supporting that intragenic deletion involving exons 2–4 might be less pathogenic for autistic symptoms.

On the other hand, U1984 who possessed a duplication-type CNV encompassing exons 3–4 manifested more severe autistic symptoms and worse cognitive function than other patients with deletion-type CNVs. The distinct phenotypes might be due to the different dosages of CNV (duplication/deletion). Interestingly, the duplication was partially overlapped with the findings from Scheuerle et al. [17]. They reported a male who had a duplication encompassing exon 2 on *PARK2* and revealed cognitive impairment and global developmental delay with particular problems on social and language skills [17]. Later, Marinai and colleagues described similar situation, and their observations also supported the idea that duplication of the *PARK2* gene possibly plays a role in the pathology of developmental anomalies [31]. Those probands with duplications from Scheuerle et al’s and Marinai et al’s studies seem to have similar clinical features with U1984 who suffered from language delay and epilepsy. Intriguingly, we observed down-regulation of EX4-5, EX6-7, and EX9-11 but normal expression of EX3-4 on the mRNA level in proband U1984 and the carrier with duplication of EX3-4 on *PARK2* in the family. Based on the expression analysis, we speculate that the copy number gain may lead to a splicing error; hence, a shorter transcript lacks most of the 3′ end of the *PARK2* mRNA (Fig. 3d). Whether the mutant transcript would interfere with the normal *PARK2* transcript (Fig. 3c) is not clear. Nevertheless, the observation that U1984 had the most severe ASD and cognitive impairment among the probands with CNVs on *PARK2* might imply that duplication of *PARK2* EX3-4 has effect beyond decreasing *PARK2* protein. These observations warrant further investigations of pathogenic mechanism of *PARK2* CNVs.

The *PARK2* gene encodes Parkin, a RING-between-RING E3 ubiquitin ligase functioning in the covalent attachment of ubiquitin to specific substrates for proteasomal degradation [32]. The structure of human Parkin ligase has been reported in exquisite detail [33–35]. Parkin is structurally tight packed by two hydrophobic interactions, which keep Parkin in an auto-inhibited conformation. A study showed that mutations (M192A, F463A, and A398T) at the RING0-RING2 domain and linker helix could disrupt the interface and lead to autoubiquitination of PARK2 (the “opening” structure) in cell [27]. The deletion encompassing exon 5 of the *PARK2* gene in U1290 and U1859 is predicted to result in in-frame deletion which leads to losing a part of RING0 domain. This may lead to a conformational change of Parkin protein and activation of the autoubiquitination. The deletion encompassing exons 6–7 of the *PARK2* gene in U1469 is predicted to cause the Glu207Leu amino acid change and lead to nonsense mutations at the 213th codon of the mutant mRNA (Additional file 1: Figure S3). We speculate that this intragenic deletion might largely influence the biological functions of Parkin owing to truncated protein lacking the C-terminal part. However, we were unable to obtain the RNA sample from the probands with deletions comprising exon 5 or exons 6–7 for gene expression analysis. Further studies to investigate how these deletions affect Parkin at the transcript level, protein level, and function level are needed to reveal *PARK2* CNV-mediated ASD pathogenesis.

Literature documents that Parkin plays a fundamental role in mitochondrial functions. The PINK1-Parkin pathway is one of well-studied pathways of mitophagy. Mutations in *PINK1* and *PARK2* are associated with neurodegeneration in Parkinson Disease [13, 25] and are hypothesized to impair the protein function and hence result in insufficient mitochondrial clearance and subsequent aggregation. In addition, a recent study has also shown that loss of Parkin function suppressed mitochondrial biogenesis through accumulation of Parkin-interacting substrate (PARIS), a transcriptional repressor of *PGC-1a,* which stimulates mitochondrial biogenesis as an activator [26]. Taken together, these evidences strongly suggest that Parkin with a loss-of-function mutation may lead to abnormal mitochondrial biogenesis and clearance, which is considered to link to the pathology of ASD [36]. In addition, Parkin protein is abundantly expressed in the human brain, including the frontal lobe cortex, substantia nigra, putamen, and locus coeruleus [28]. Therefore, with the observations reported in this study, we suspect that the ASD-associated *PARK2* CNV might contribute to the pathogenesis of ASD through affecting mitochondria in particular brain region (e.g., frontal lobe) in a proportion of children.

## Conclusions

In summary, our study indicated that *PARK2* could be a pathogenic gene associated with ASD in the Han Chinese population. With limited samples, we also observed that exonic duplication might result in a more severe interference of *PARK2* expression and the clinical feature than deletion at the A region (exons 2–4) of the *PARK2* locus*.* The intriguing results of this work warrant further study on characterizing the functional impact of various exonic CNVs on the *PARK2* gene and its role in mitochondria malfunction in the brain of patients with ASD.

## Additional file

Additional file 1: The detailed methods of experiments, clinical information and more experimental results. (DOCX 1271 kb)

## Abbreviations

aCGH: array-based comparative genomic hybridization
ADHD: attention-deficit/hyperactivity disorder
ADI-R: Autism Diagnostic Interview-Revised
AQ: autism-spectrum quotient
ASD: autism spectrum disorders
cDNA: complementary deoxyribonucleic acid
CNV: copy number variations
DNA: deoxyribonucleic acid
DSM-IV: Diagnostic and Statistical Manual of Mental Disorders, Fourth Edition
EX: exon
GWAS: genome-wide association study
HFA: high-function autism
IQ: intelligence quotients
mRNA: messenger ribonucleic acid
NCGM: National Center for Genome Medicine
PARIS: Parkin-interacting substrate
PCR: polymerase chain reaction
PD: Parkinson disease
qRT-PCR: quantitative reverse transcriptase polymerase chain reaction
RNA: ribonucleic acid
SNP: single nucleotide polymorphism
SRS: Social Responsiveness Scale
WCST: Wisconsin Card Sorting Test
WISC-III: Weschler Intelligence Scale for Children-3^rd^ edition
