# Supplementary material for: Genome-wide analysis of copy number variations identifies PARK2 as a candidate gene for autism spectrum disorder
Source: Mol Autism. 2016 Apr 1;7:23. doi: 10.1186/s13229-016-0087-7 (PMC4818409; doi:10.1186/s13229-016-0087-7)
Supplement: Additional file 1: — The detailed methods of experiments, clinical information and more experimental results. (DOCX 1271 kb) [file 13229_2016_87_MOESM1_ESM.docx]

**CONTENTS**

[A. GENOTYPING AND DATA CLEANING ……………………………………………………………………..4](#_Toc443343017)

[Genotyping and sample quality control …………………………………………………………………………..4](#_Toc443343018)

[Intensity data analysis and CNV extraction …………………………………………………………………….5](#_Toc443343019)

[Measurement of global CNV burden ……………………………………………………………………………..6](#_Toc443343020)

[B. BEHAVIOR PHENOTYPE AND NEUROCOGNITIVE ENDOPHENOTYPE MEASUREMENTS ………………………………………………………………………………………………………7](#_Toc443343021)

[The Autism Diagnostic Interview-Revised (ADI-R) ……………………………………………………..7](#_Toc443343022)

[Social Reciprocity Scale (SRS) ……………………………………………………………………………………..8](#_Toc443343023)

[Weschler Intelligence Scale for Children-3rd edition (WISC-III)………………………. 8](#_Toc443343024)

[Wisconsin Card Sorting Test (WCST) ……………………………………………………………………………9](#_Toc443343025)

[C. SUPPLEMENTARY FIGURES AND TABLES ………………………………………………………….11](#_Toc443343026)

[D. SUPPLEMENTARY REFERENCES ………………………………………………………………………….48](#_Toc443343027)

**LIST OF FIGURES**

[Supplementary figure S1. The distribution of CNV load and CNVcount in the screen cohort.. 12](#_Toc443343242)

[Supplementary figure S2. Flowchart of identification of case-specific CNV segments and CNV loci………………………………………………………………. 14](#_Toc443343244)

[Supplementary figure S3. Validation of two duplications in two healthy control ……………..15](#_Toc443343245)

[Supplementary figure S4. Validation of exons6-7 deletion in U1469 …………………………17](#_Toc443343246)

**LIST OF TABLES**

[Supplementary Table S1. Statistics of CNV burden …………………………………………...19](#_Toc443343305)

[Supplementary Table S2. Information of primers ……………………………………………..20](#_Toc443343306)

[Supplementary Table S3. List of case-specific CNV loci ……………………………………..22](#_Toc443343307)

[Supplementary Table S4. Six well known ASD-associated CNV loci ………………………..44](#_Toc443343308)

[Supplementary Table S5. Summary of CNV findings at the *PARK2* locus ……………………46](#_Toc443343309)

[Supplementary Table S6. Autism-Spectrum Quotient and Adult Self-Report Inventory-IV… 47](#_Toc443343310)

1. GENOTYPING AND DATA CLEANING

Genotyping and sample quality control

Initially, 350 ASD cases were genotyped with the Affymetrix Genome-Wide Human SNP Array 6.0 in four batches due to the long period of sample collection. The SNP array contains more than 906,600 probes for single nucleotide polymorphisms (SNPs) and more than 946,000 non-polymorphic probes for copy number variations (CNVs). Total 202,000 selected CNV probes were based on known reported copy number changes (Database of Genomic Variants, Toronto) and 744,000 were selected for their spacing. Genotype calling was done using Genotyping Console Version 4.1 (Affymetrix, CA, USA). The average call rate was 99.49 + 0.29 % (ranging from 97.83 % to 99.88 %) and all samples passed genotyping quality control (call rate >= 95%) . Computed gender was called based on cn-probe- chrXY-ratio_gender method from Affymetrix Power Tools (Affymetrix, CA, USA). Samples with mismatched gender between computed gender and case information were excluded from analysis. Duplicated samples detected by Kinship analysis using P-Link software were also excluded. In total, 15 ASD cases were excluded according to sample QC.

A total of 1111 individuals from the Han-Chinese Cell and Genome Bank in Taiwan that were genotyped with the same SNP array in the same four batches of the ASD subjects were selected to serve as disease-free controls. Healthy control samples with (1) genotype call rate < 95 %, (2) aneuploidy, or (3) number of CNV regions exceeded the 3 SD of the mean were excluded from association analysis. In total, 18 healthy controls were excluded according to sample QC filter.

Finally, a total of 335 ASD cases and 1093 healthy controls were included for the association analysis.

Intensity data analysis and CNV extraction

Copy number estimation for cases and controls genotyped in the same batch was carried out with Genotyping Console Version 4.1 (Affymetrix, CA, USA) under unpaired mode where the corresponding healthy controls were used as the reference set. Since it was CNV regions rather than SNP markers that were subjected to association analysis, individual SNP quality was not considered as a filter factor for copy number variant (CNV) prediction. Regions that contained at least twenty consecutive probes with the same direction of copy number change were defined as having CNVs. CNV regions overlapped with centromeric regions (hg19, UCSC), antibody variable regions (PennCNV, http://www.openbioinformatics.org/penncnv/penncnv_faq.html#ig) and T-cell receptor loci (NCBI Gene, http://www.ncbi.nlm.nih.gov/gene/) were filtered out prior to association analysis.

Measurement of global CNV burden

Only the CNV regions past the above-mentioned filter and located on autosomes were subjected to measurement of global CNV burden, including total length of CNVs and total counts of CNVs. The total length of CNVs is the total base pairs of altered copy number in each individual, i.e. CNV load. The CNV count is the total number of CNV regions in each individual. The analysis was also stratified by deletion-type and duplication-type CNVs. Two-tailed Two-sample t-test was performed to determine the association of global CNV load and count with autism subjects.

There was a significant increase of total CNV load associated with autism subjects (Supplementary Figure S1a and Supplementary Table S1). Interestingly the elevated CNV load is specific to duplication-type CNV but not seen for deletion-type CNV (Supplementary Figure S1b and S1c and Supplementary Table S1). This result is consistent with the report by Girirajan et al., who showed that there was a global increase in duplicated base pairs associated with autism [[1](#_ENREF_1)]. We also observed that the counts of CNVs in cases were significantly higher than that in controls (Supplementary Figure S1d and Supplementary Table S1). The difference was independent of CNV type, yet the fold change of counts of deletion-type CNVs was much less than that of duplication-type CNVs (1.15 vs. 1.37) (Supplementary Figure S1e and S1f and Supplementary Table S1). Although this result is a little bit surprising, it is understandable given that higher global CNV burden is associated with autism.

1. BEHAVIOR PHENOTYPE AND NEUROCOGNITIVE ENDOPHENOTYPE MEASUREMENTS

The Autism Diagnostic Interview-Revised (ADI-R)

The ADI-R is a standardized, comprehensive, semi-structured, investigator-based interview covering most developmental and behavioral aspects of ASD [[2](#_ENREF_2)]. It is administered to the child’s caregiver. Diagnostic assignment is made following a diagnostic algorithm for the DSM-IV (American Psychiatric Association 1993) and the ICD-10 definition of autism (World Health Organization (WHO), 1992). The ADI-R is appropriate for interviewing caregivers of children with a mental age from about 18 months into adulthood. The ADI-R requires approximately 2 to 3 hours to complete with the caregiver and typically is videotaped for later scoring. The scoring yields summary scores in the following domains: qualitative impairments in reciprocal social interaction, communication, and repetitive behaviors and stereotyped patterns. Cut-off scores are available for making the diagnosis of autism versus individuals without a diagnosis of autism. The Chinese version of the ADI-R, translated into Chinese by Gau and colleagues, has been approved by the Western Psychological Services in 2007, and is widely used in ASD research [[3-6](#_ENREF_3)].

Social Reciprocity Scale (SRS)

The SRS is a 65-item questionnaire that inquires about the child’s social interactions with others [[7](#_ENREF_7)]. Thirty-five items are directly related to reciprocal social behavior (criterion “a” for autistic disorder), 6 items are related to language deficits (criterion “b”), 20 items represent criterion “c”, and 4 items inquire about miscellaneous symptoms. All items are rated on a scale from 0 (not true) to 3 (almost always true), based on the frequency of the behavior. The SRS can be completed by a parent or other adults (e.g., teachers) who routinely observes the child’s social interactions with peers and adults in 15-20 minutes. The study compared the SRS with the Autism Diagnostic Interview-Revised (ADI-R) in 61 child psychiatric patients. The SRS is highly correlated with ADI-R algorithm scores for DSM-IV criterion and exhibited good inter-rater reliability [[8](#_ENREF_8)]. The SRS was translated to Chinese by Gau and colleagues [[9](#_ENREF_9)]. The Chinese version of the SRS has been widely used in community-based and clinic-based studies. The SRS has been shown to provide a continuous measure of social disability that has important implications for genetic research [[10](#_ENREF_10)].

Weschler Intelligence Scale for Children-3rd edition (WISC-III)

Weschler Intelligence Scale for Children-3rd edition (WISC-III, Wechsler, 1991) has been widely used to assess full-scale intelligence levels of children aged 6 years to 16 years, 11 months. WISC-III is composed of 13 subtests to test children’s cognitive ability of different dimensions, which are grouped into two scores: performance IQ score and verbal IQ score. Performance IQ score (7 subtests) includes Picture Completion, Block Design, Object Assembly, Picture Arrangement, Coding, Symbol Search and Mazes subtests. Verbal IQ score (6 subtests) includes Information, Comprehension, Arithmetic, Similarities, Digit Span and Vocabulary subtests. Factor analysis has been conducted to demonstrate the groupings other than the simple Verbal/Performance IQ. Four composite subscales have been created: (1) Verbal Comprehension: Information, Similarities, Vocabulary, and Comprehension; (2) Perceptual Organization: Picture Completion, Picture Arrangement, Block Design, and Object Assembly; (3) Freedom From Distractibility: Arithmetic and Digit span; and (4) Process Speed: Coding and Symbol Search [[11](#_ENREF_11)].

Wisconsin Card Sorting Test (WCST)

The Wisconsin Card Sorting Test (WCST) was used to assess the ability to form abstract concepts, and shift and maintain the set. The test was developed to assess abstraction ability. This test provides information about several aspects of problem solving behaviors beyond such basic indices of task success or failure. Examples of such indices include the number of perseverance responses, the number of perseverative errors, the failure to maintain the set, and the number of categories achieved. Moreover, the WCST is especially sensitive to lesions of the frontal lobe. The test consists of four stimulus cards, placed in front of the subject, the first with a red triangle, the second with two green stars, the third with three yellow crosses, the fourth with four blue circles on them. The subject is then given two packs, each containing 64 response cards, which have designs similar to those on the stimulus cards, varying in color, geometric form, and number. The subject was instructed to match each of the cards in the decks to one of the four key cards. The examiner explained that the object is to try to get as many correct responses as possible and that there will be no time limit to this test. The subject was told each time whether he or she is right or wrong. The WCST was used to assess the mental flexibility in child participants with ASD [[12-14](#_ENREF_12)].

1. SUPPLEMENTARY FIGURES AND TABLES

1. The distribution of CNV load and CNVcount in the screen cohort
2.
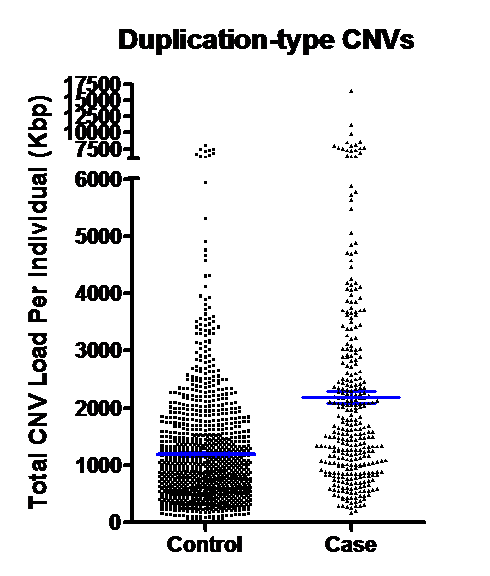

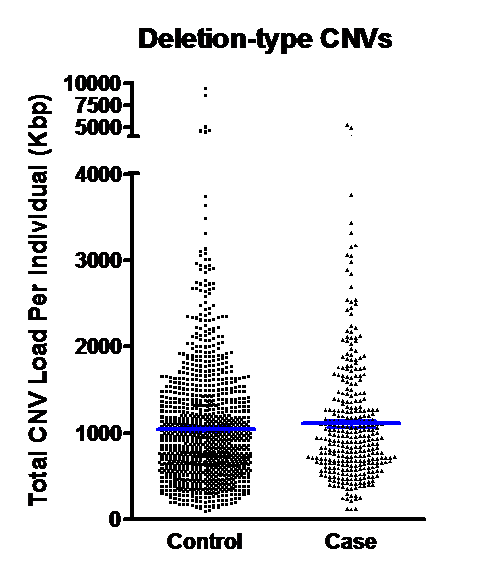

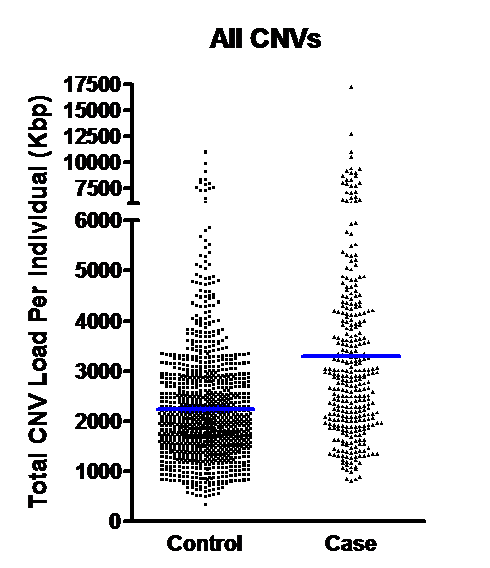
 **b. c.**


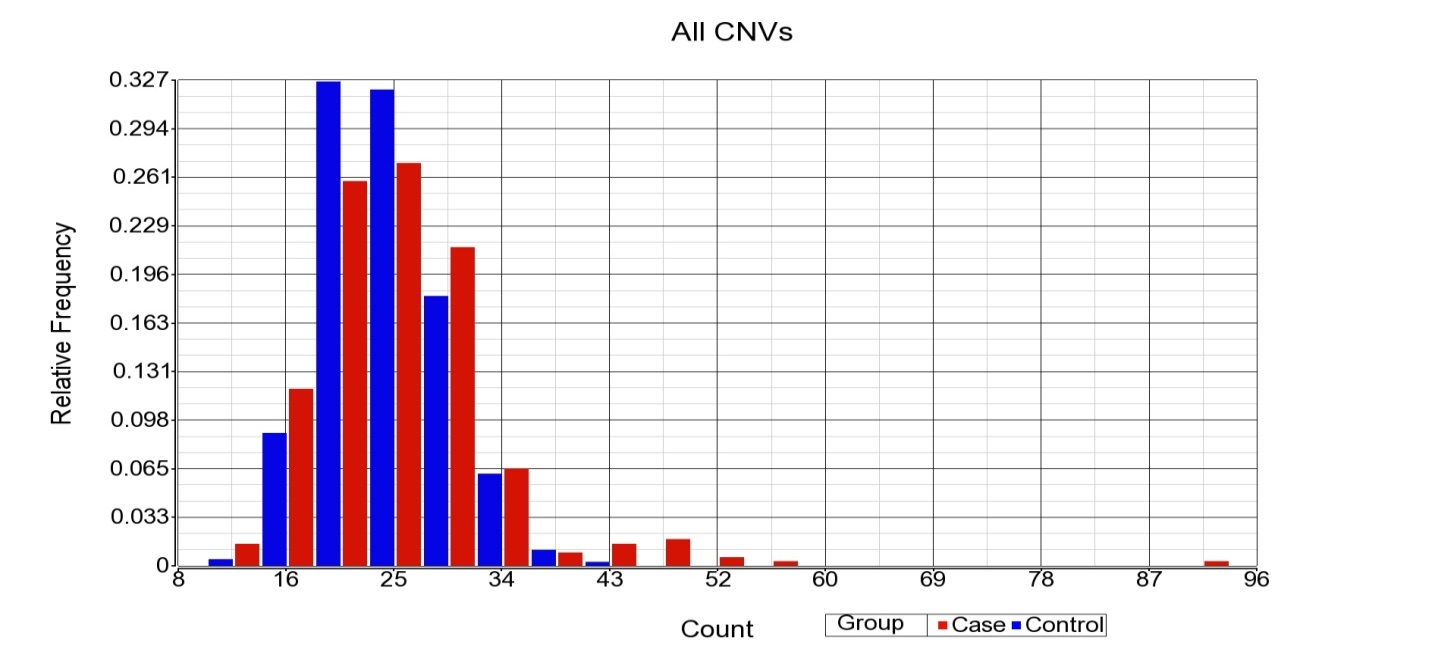
**d.**

**e.**


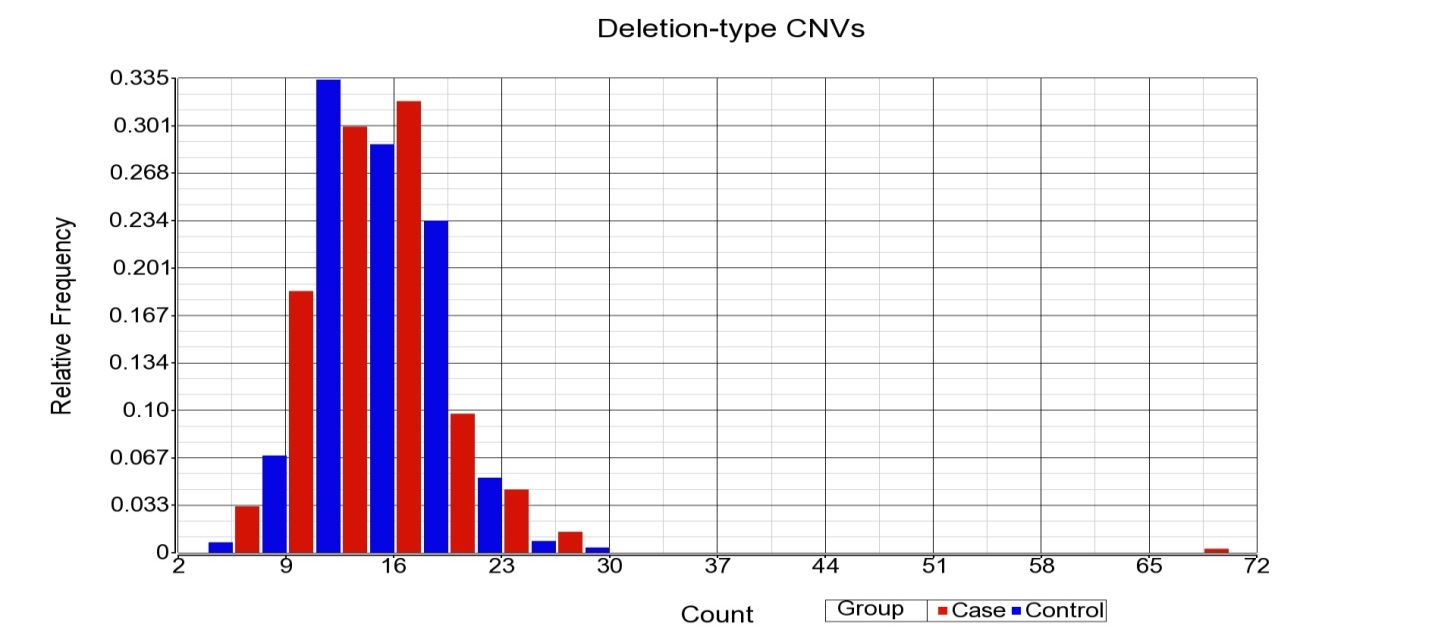


**f.**


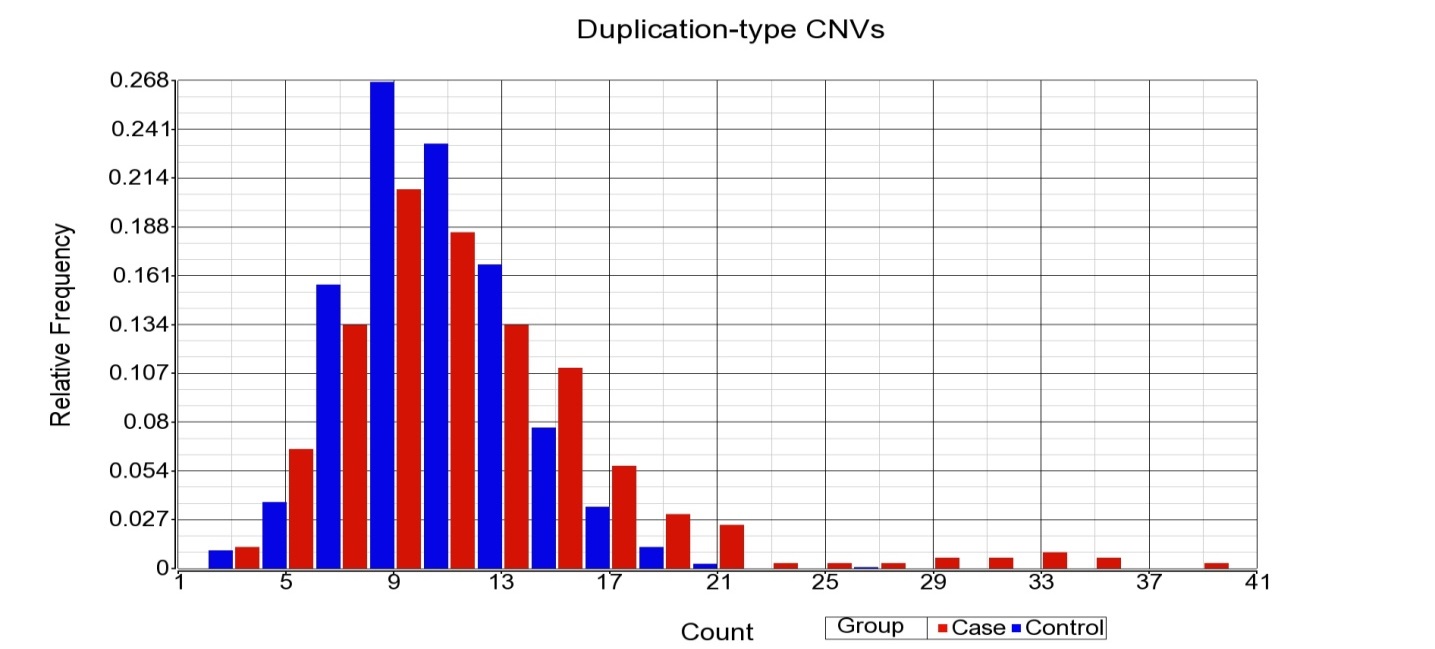


Distributions of CNV load and CNV counts for ASD subjects and control subjects in the screen cohort. Distributions of CNV load (Mbp) of (a) all CNV regions, (b) deletion-type CNVs, and (c) duplication-type CNVs for each individual are shown. Each dot represents one individual and horizontal lines denote the mean ± SEM. Relative frequency distributions of CNV counts of (d) all CNV regions, (e) deletion-type CNVs, and (f) duplication-type CNVs per individual are shown. The height of a bar indicates the relative frequency of subjects with the value of counts in a given bin. Red bars designate ASD subjects while blue bars designate control subjects.

1. Flowchart of identification of case-specific CNV segments and CNV loci.


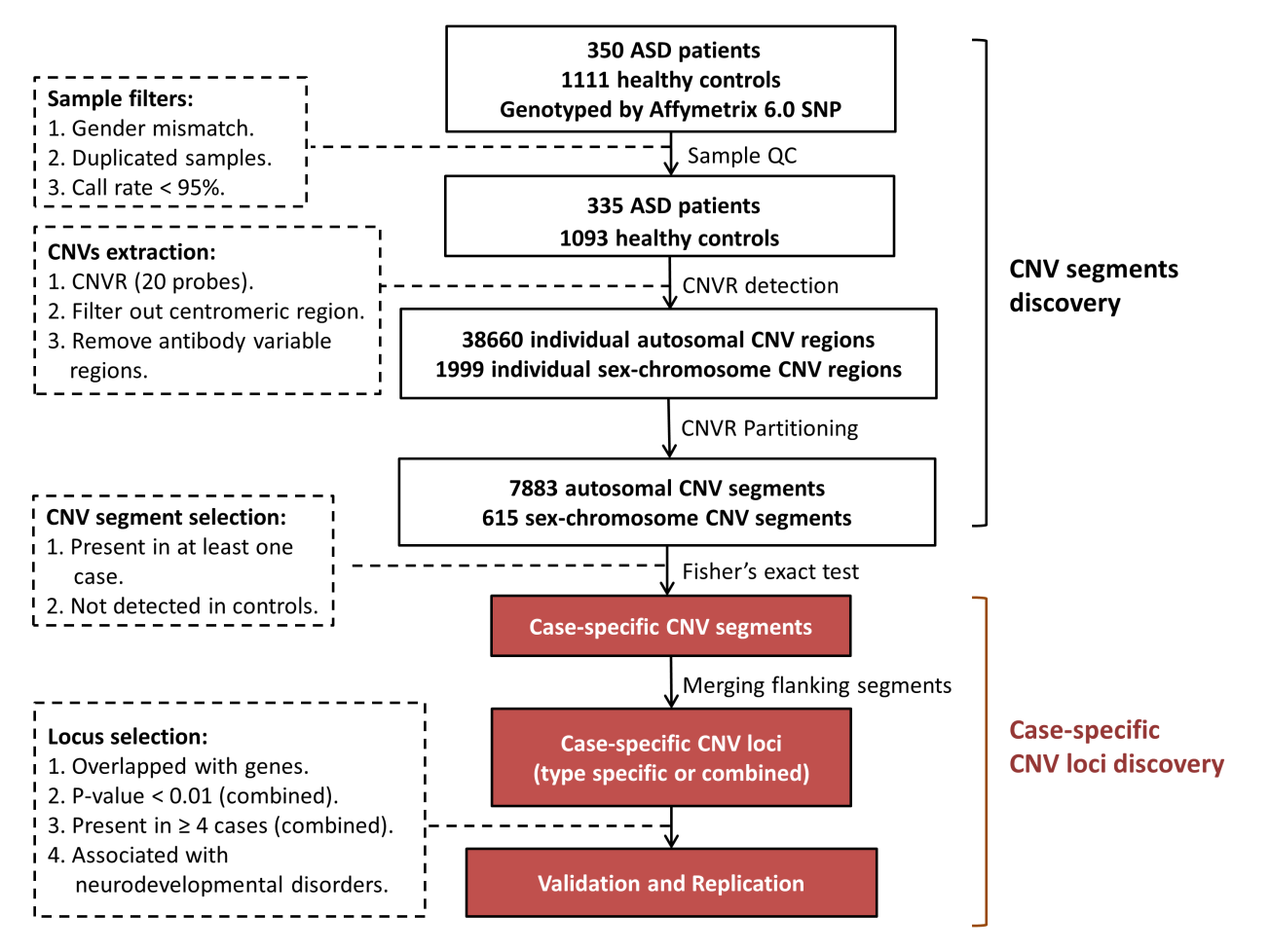


1.
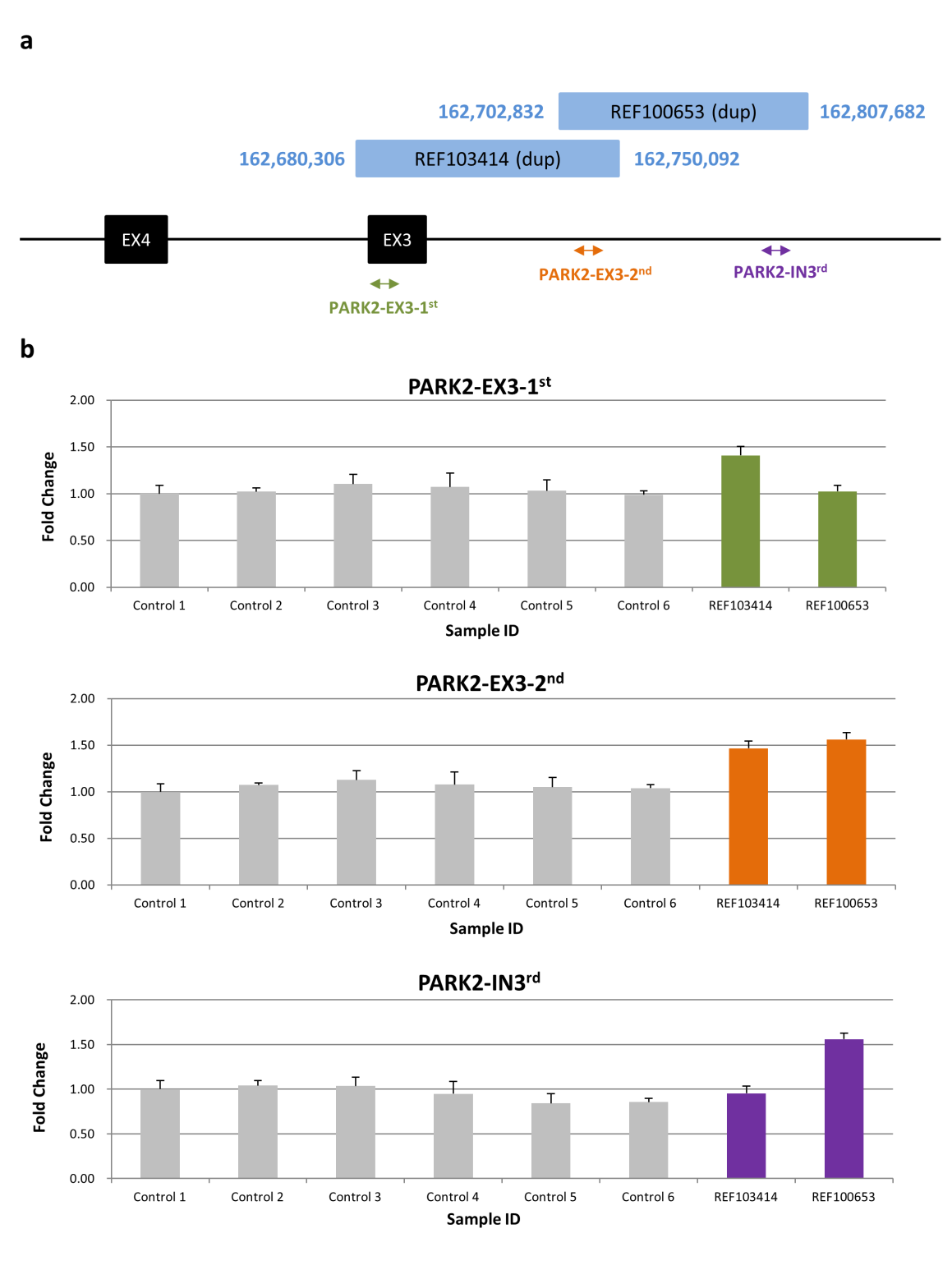
Validation of two duplications in two healthy control

Validation of duplications within A region at the *PARK2* locus in two healthy controls (REF103414 and REF100653). (a) Location of the predicted CNV regions and the three pairs of primers used for genomic qPCR in accordance with the *PARK2* gene. EX, exon. (b) Results of genomic qPCR. Gray bar represented the controls without CNV at the *PARK2* gene locus. Green, orange and purple bar represented the two healthy controls with duplication at the *PARK2* gene locus. Error bars denote the SD of triplicate runs.

1. Validation of exons6-7 deletion in U1469


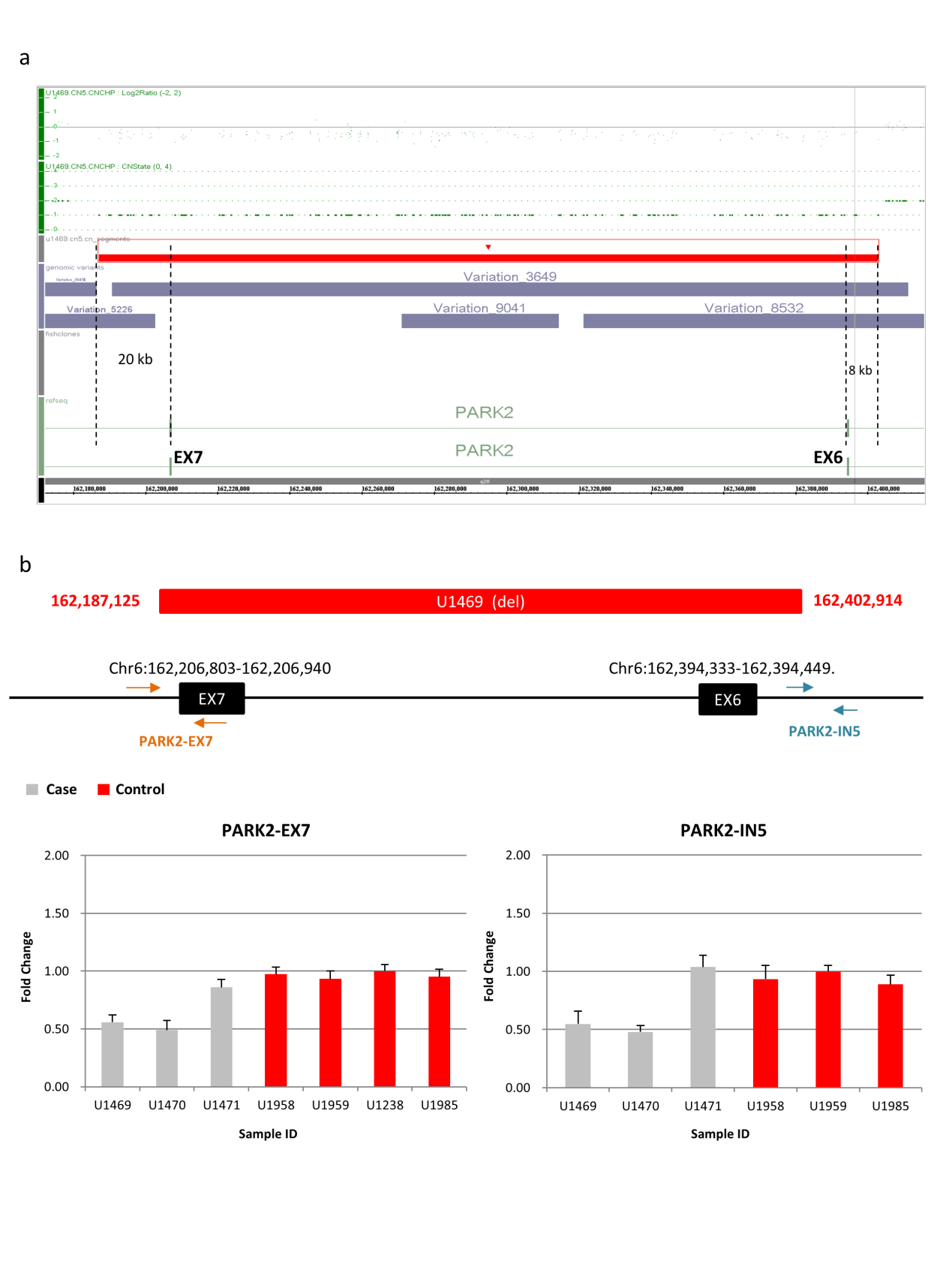


Deletion in U1469 covering exons 6 and 7 at the *PARK2* locus. (a) Genomic location of CNV at 6q26 in U1469 based on SNP genotyping data. Log2 Ratio and inferred CN State of probes are presented. Horizontal red bar indicate the predicted CNV region. Genomic coordinates corresponds to human genome Build 37 (hg 19). EX, exon. (b) Location of the two pairs of primers used for genomic qPCR in accordance with the predicted CNV region and the *PARK2* gene. (c) Results of genomic qPCR. Gray bar represented the ASD cases and red bar represented the controls. Error bars denote the SD of triplicate runs.

1. Statistics of CNV burden

| CNV burden | Case (n=335)* | Control (n=1093)* | p-value  (Case vs. Control) | FoldChange  (Case vs. Control) |
| --- | --- | --- | --- | --- |
| Total Load (Kbp) | 3295.54 ± 2019.03 | 2237.98 ± 1171.57 | <0.0001 | 1.47 |
| Dup_Load (Kbp) | 2183.07 ± 1968.43 | 1190.15 ± 996.72 | <0.0001 | 1.83 |
| Del_Load (Kbp) | 1112.47 ± 722.19 | 1047.84 ± 723.05 | 0.2251 | 1.06 |
| Total Count | 28.08 ± 7.78 | 22.64 ± 4.71 | <0.0001 | 1.24 |
| Dup_Count | 12.41 ± 5.48 | 9.06 ± 3.08 | <0.0001 | 1.37 |
| Del_Count | 15.67 ± 5.10 | 13.58 ± 3.73 | <0.0001 | 1.15 |

*The data is presented as mean ± SD.

1. Information of primers

| **Primer pair name** | **Forward primer (5’→3’)** | **Reverse primer (5’→3’)** | **Tm (℃)** | **Size (bp)** | **Application** | |
| --- | --- | --- | --- | --- | --- | --- |
| PARK2-EX3 | GCCCCAGTTCAGTGTTGTTT | CTTTTCTCCACGGTCTCTGC | 60 | 249 | CNV validation (initial stage), for A region screening |  |
| PARK2-EX5 | TTTCCCAAAGGGTCCATCTT | ACTAGTCCCAGGGCAGTGTG | 60 | 91 | CNV validation (initial stage), for B region screening |  |
| PARK2-EX7 | CAGTTTGTTCACGACCCTCA | CAATTCCTTCATTCCCCAGA | 60 | 230 | CNV validation (initial stage), for C region screening |  |
| PARK2-IN5 | TCCCTCGCTTAAAGAGCAAA | TGTTTTAGAAGGGCCCTGAG | 60 | 53 | CNV validation (initial stage) |  |
| PARK2-EX3-1^st^ | CCAGTCTGCTTTGAACACTTTTT | TCAGATTGAACTCGTGATGTATTTTA | 60 | 200 | CNV validation in controls (initial stage) |  |
| PARK2-EX3-2^nd^ | TGTCCAGTTGTTTTTACTTCTTGCT | TGCACTCATAGTTTTCTTATCTTCAGTT | 60 | 148 | CNV validation in controls (initial stage) |  |
| PARK2-IN3^rd^ | GAATGAGCCAGAGTTCAGATAAAGATA | ATGCCTTAGGGCTTGTAGTTTCTTA | 60 | 150 | CNV validation in controls (initial stage) |  |
| PARK2-EX1 | CTGCCAGGTACAGCCTCTCT | GGTCTTCATGAGAACGCTCAG | 60 | 231 | Confirm the coverage of CNV at *PARK2* locus |  |
| PARK2-EX2 | CCTTCCAATTTCCTTGGTCA | CACCTGCACAGTCCAGTCAT | 60 | 188 | Confirm the coverage of CNV at *PARK2* locus |  |
| PARK2-EX4 | GCTTTTTCTTCTCCAGCAGGT | CATGCTGACACTGCATTTCC | 60 | 162 | Confirm the coverage of CNV at *PARK2* locus |  |
| FARP2 | AATGCGATGGCCAGGTATTA | ATGAAAGATCTTGCGGCTGT | 60 | 172 | Internal control for A region |  |
| GAPDH | TGCCTTCTTGCCTCTTGTCT | GGCTCACCATGTAGCACTCA | 60 | 146 | Internal control for B, C regions and CNV validation |  |
| PARK2 EX3-4 | ACCTCAGCAGCTCAGTCCTC | TGCTGCACTGTACCCTGAGT | 60 | 186 | For *PARK2* expression analysis (CNV-affected region) |  |
| PARK2 EX4-5 | AAAGGCCCCTGTCAAAGAGT | ACTAGTCCCAGGGCAGTGTG | 60 | 165 | For *PARK2* expression analysis (CNV-affected region) |  |
| PARK2 EX6-7 | CGCAACAAATAGTCGGAACA | AAGGCAGGGAGTAGCCAAGT | 60 | 179 | For *PARK2* expression analysis (CNV-unaffected region) |  |
| PARK2 EX9-11 | GCTGTGGGTTTGCCTTCTG | GGTTTCTTTGGAGGCTGCTT | 60 | 170 | For *PARK2* expression analysis (CNV-unaffected region) |  |
| GAPDH EX1-2 | CTCTGCTCCTCCTGTTCGACA | ACGACCAAATCCGTTGACTC | 60 | 112 | For *PARK2* expression analysis (internal control) |  |

1. List of case-specific CNV loci

| **Chr** | **CN segment (start)** | **CN segment (stop)** | **Size**  **(bp)** | **Gene symbol** | **Cases (n=335)** | **CNVs type** | **Peak Fisher_P** |
| --- | --- | --- | --- | --- | --- | --- | --- |
| 1 | 904595 | 1130727 | 226133 | *PLEKHN1,C1orf170,HES4,ISG15,AGRN,AK310350,BC033949,RNF223,C1orf159,LOC254099,MIR200B,JA715134,MIR200A,JA715143,MIR429,AK128833,TTLL10* | 1 | gain | 0.235 |
| 1 | 12834253 | 12845851 | 11599 | *PRAMEF12* | 1 | gain | 0.235 |
| 1 | 13377035 | 13538299 | 161265 | *PRAMEF8,PRAMEF9,PRAMEF13,PRAMEF19,PRAMEF16,PRAMEF20* | 6 | gain | <0.001 |
| 1 | 17262235 | 17295200 | 32966 | *CROCC* | 4 | gain | 0.003 |
| 1 | 25611440 | 25673142 | 61703 | *AX747205,RHD,C1orf63,TMEM50A* | 1 | gain | 0.235 |
| 1 | 36422543 | 36475394 | 52852 | *AGO3* | 1 | gain | 0.235 |
| 1 | 54978948 | 55303782 | 324835 | *ACOT11,FAM151A,MROH7,HEATR8-TTC4,TTC4,PARS2,TTC22,C1orf177* | 2 | gain | 0.055 |
| 1 | 71137136 | 71424426 | 287291 | *BC041441,PTGER3* | 1 | gain | 0.235 |
| 1 | 82134942 | 82211753 | 76812 | *LPHN2* | 1 | gain | 0.235 |
| 1 | 94270328 | 94315043 | 44716 | *MIR760,BCAR3,TRNA_Arg* | 1 | gain | 0.235 |
| 1 | 94487354 | 94574931 | 87578 | *ABCA4* | 1 | gain | 0.235 |
| 1 | 95155585 | 95156242 | 658 | *BC030750* | 1 | gain | 0.235 |
| 1 | 104268210 | 104404832 | 136623 | *AMY1A* | 1 | gain | 0.235 |
| 1 | 108828945 | 109145069 | 316125 | *NBPF4,BC051808,NBPF6,FAM102B* | 2 | gain | 0.055 |
| 1 | 142569069 | 144007037 | 1437969 | *DQ579288,DQ586768,DQ583161,DQ590589,ANKRD20A12P,BC071797,DQ590126,DQ592442,BC053679,BC029473,AK056396,CR936796,DQ587539,DQ596206,BC070106,TRNA_Asn,DQ571491,LOC100130000,LINC00875,PPIAL4G,FAM72D,SRGAP2B* | 2 | gain | 0.055 |
| 1 | 144991278 | 145290292 | 299015 | *LOC653513,PDE4DIP,BC065231,BX647792,SEC22B,NOTCH2NL,NBPF10,LOC100288142,NBPF9* | 2 | gain | 0.055 |
| 1 | 149732613 | 149772497 | 39885 | *FCGR1A,AX747534,HIST2H2BF* | 1 | gain | 0.235 |
| 1 | 157931997 | 158068478 | 136482 | *KIRREL,hCG_1995134* | 1 | gain | 0.235 |
| 1 | 203654395 | 203665861 | 11467 | *ATP2B4* | 1 | gain | 0.235 |
| 1 | 227992928 | 228045990 | 53063 | *PRSS38* | 1 | gain | 0.235 |
| 1 | 240298870 | 240307389 | 8520 | *FMN2* | 1 | gain | 0.235 |
| 1 | 22020152 | 22088060 | 67909 | *USP48* | 1 | loss | 0.235 |
| 1 | 25105256 | 25178319 | 73064 | *CLIC4,Z24749* | 1 | loss | 0.235 |
| 1 | 25583329 | 25595935 | 12607 | *C1orf63* | 1 | loss | 0.235 |
| 1 | 53487858 | 53498724 | 10867 | *SCP2* | 1 | loss | 0.235 |
| 1 | 59126414 | 59141365 | 14952 | *MYSM1* | 1 | loss | 0.235 |
| 1 | 145605318 | 145625694 | 20377 | *POLR3C,RNF115,LOC100288142,NBPF10* | 1 | loss | 0.235 |
| 1 | 160492270 | 160675090 | 182821 | *SLAMF6,CD84,SLAMF1,CD48* | 1 | loss | 0.235 |
| 1 | 171048847 | 171340430 | 291584 | *FMO3,MIR1295B,MIR1295A,FMO6P,FMO2,Y_RNA,FMO1,TOP1P1,FMO4* | 1 | loss | 0.235 |
| 1 | 174802140 | 174802829 | 690 | *RABGAP1L* | 4 | loss | 0.003 |
| 1 | 180332687 | 180385459 | 52773 | *ACBD6* | 1 | loss | 0.235 |
| 1 | 189991289 | 190699301 | 708013 | *FAM5C,CR936711,LOC440704* | 1 | loss | 0.235 |
| 1 | 196909633 | 196916787 | 7155 | *CFHR2* | 1 | loss | 0.235 |
| 1 | 225150542 | 225205321 | 54780 | *DNAH14* | 1 | loss | 0.235 |
| 1 | 231760894 | 231764132 | 3239 | *TSNAX-DISC1,DISC1* | 1 | loss | 0.235 |
| 1 | 245959395 | 245960306 | 912 | *SMYD3* | 1 | loss | 0.235 |
| 2 | 263073 | 374086 | 111014 | *SH3YL1,ACP1,FAM150B* | 1 | gain | 0.235 |
| 2 | 43416536 | 43466619 | 50084 | *ZFP36L2,LOC100129726,THADA* | 1 | gain | 0.235 |
| 2 | 58164223 | 59360403 | 1196181 | *VRK2,FANCL,FLJ30838* | 1 | gain | 0.235 |
| 2 | 74857355 | 75324605 | 467251 | *M1AP,SEMA4F,HK2,TRNA_Glu,AK125960,POLE4,MIR5000,TACR1* | 1 | gain | 0.235 |
| 2 | 87161841 | 87330966 | 169126 | *RGPD1,PLGLB2,LOC285074,RMND5A* | 1 | gain | 0.235 |
| 2 | 91801473 | 92136779 | 335307 | *LOC654342,Mir_544,GGT8P,ACTR3BP2* | 7 | gain | <0.001 |
| 2 | 96236590 | 96422939 | 186350 | *TRIM43,BC016831* | 1 | gain | 0.235 |
| 2 | 125532806 | 126740629 | 1207824 | *CNTNAP5* | 2 | gain | 0.055 |
| 2 | 131970783 | 132028905 | 58123 | *POTEE,PLEKHB2* | 1 | gain | 0.235 |
| 2 | 132417251 | 132458677 | 41427 | *BX648270* | 1 | gain | 0.235 |
| 2 | 132881501 | 133117248 | 235748 | *ANKRD30BL,MIR663B,JA668105,AK094599* | 3 | gain | 0.013 |
| 2 | 137319708 | 137598039 | 278332 | *THSD7B* | 1 | gain | 0.235 |
| 2 | 160048257 | 160081336 | 33080 | *TANC1* | 1 | gain | 0.235 |
| 2 | 160279324 | 160408219 | 128896 | *BAZ2B* | 1 | gain | 0.235 |
| 2 | 233790821 | 233801460 | 10640 | *NGEF* | 1 | gain | 0.235 |
| 2 | 11318639 | 11386971 | 68333 | *PQLC3,ROCK2* | 1 | loss | 0.235 |
| 2 | 24026668 | 24078728 | 52061 | *ATAD2B* | 1 | loss | 0.235 |
| 2 | 29363803 | 29393250 | 29448 | *CLIP4* | 1 | loss | 0.235 |
| 2 | 29577405 | 29597044 | 19640 | *ALK* | 1 | loss | 0.235 |
| 2 | 38789508 | 38832850 | 43343 | *HNRPLL* | 1 | loss | 0.235 |
| 2 | 40574269 | 40576095 | 1827 | *SLC8A1* | 1 | loss | 0.235 |
| 2 | 42031326 | 42134336 | 103011 | *Y_RNA,U4,LOC388942* | 1 | loss | 0.235 |
| 2 | 71560665 | 71591970 | 31306 | *ZNF638* | 1 | loss | 0.235 |
| 2 | 87337957 | 87344726 | 6770 | *TRNA_Pseudo,RMND5A* | 1 | loss | 0.235 |
| 2 | 99255901 | 99306504 | 50604 | *MGAT4A* | 1 | loss | 0.235 |
| 2 | 129497534 | 130166044 | 668511 | *AK311291,LOC151121* | 1 | loss | 0.235 |
| 2 | 130197500 | 131211699 | 1014200 | *LOC389033,LOC100131320,RAB6C,LOC440905,DQ573684,DQ587539,DQ582260,DQ595048,DQ589348,DQ590589,POTEF,CCDC74B-AS1,CCDC74B,SMPD4,FLJ14346,MZT2B,TUBA3E,TRNA,TRNA_Pseudo,TRNA_Ser,TRNA_Glu,CCDC115,IMP4,PTPN18,LOC100216479,BC069809* | 1 | loss | 0.235 |
| 2 | 142342699 | 142419722 | 77024 | *LRP1B* | 1 | loss | 0.235 |
| 2 | 179100599 | 179152215 | 51617 | *SNORD77,OSBPL6* | 1 | loss | 0.235 |
| 2 | 214325049 | 214339834 | 14786 | *SPAG16* | 1 | loss | 0.235 |
| 2 | 228560664 | 228610384 | 49721 | *AX746677,SLC19A3* | 1 | loss | 0.235 |
| 2 | 238318241 | 242941546 | 4623306 | *COL6A3,MLPH,PRLH,RAB17,LRRFIP1,RBM44,RAMP1,UBE2F,UBE2F-SCLY,SCLY,ESPNL,KLHL30,FAM132B,ILKAP,LOC151174,LOC643387,HES6,PER2,TRAF3IP1,AF055024,ASB1,LOC151171,U4,TWIST2,FLJ43879,MIR4440,MIR4441,HDAC4,MGC16025,MIR4269,MIR2467,BC132948,LOC150935,MIR4786,NDUFA10,OR6B2,PRR21,OR6B3,MYEOV2,OTOS,MIR149,PP14571,GPC1,ANKMY1,DUSP28,RNPEPL1,CAPN10,GPR35,AQP12B,AQP12A,KIF1A,AGXT,C2orf54,LOC200772,AK055890,SNED1,MTERFD2,PASK,PPP1R7,ANO7,DKFZp686L08115,HDLBP,2-Sep,AK055601,FARP2,STK25,BOK-AS1,BC017214,BOK,5S_rRNA,THAP4,ATG4B,AK027332,AK126180,DTYMK,ING5,D2HGDH,GAL3ST2,PABL,NEU4,PDCD1,CXXC11,AK097934* | 1 | loss | 0.235 |
| 3 | 2773571 | 2880210 | 106640 | *CNTN4* | 1 | gain | 0.235 |
| 3 | 10121423 | 10142452 | 21030 | *FANCD2,FANCD2OS* | 1 | gain | 0.235 |
| 3 | 10332064 | 10382719 | 50656 | *GHRLOS,GHRL,SEC13,ATP2B2* | 1 | gain | 0.235 |
| 3 | 19325476 | 19396453 | 70978 | *KCNH8,MIR4791* | 1 | gain | 0.235 |
| 3 | 41738981 | 41795966 | 56986 | *ULK4* | 1 | gain | 0.235 |
| 3 | 75639495 | 75930920 | 291426 | *MIR1324,FLJ20518,LOC401074,ZNF717,MIR4273* | 10 | gain | <0.001 |
| 3 | 77251183 | 77307272 | 56090 | *ROBO2* | 1 | gain | 0.235 |
| 3 | 79440654 | 79696408 | 255755 | *ROBO1* | 1 | gain | 0.235 |
| 3 | 82244675 | 82247295 | 2621 | *BC031255* | 1 | gain | 0.235 |
| 3 | 128220875 | 128367564 | 146690 | *LOC90246,C3orf27,RPN1* | 1 | gain | 0.235 |
| 3 | 128483300 | 128665790 | 182491 | *RAB7A,DQ583119,DQ598665,DQ570601,DQ579267,DQ572301,DQ576681,LOC653712,ACAD9,AK125726,KIAA1257* | 1 | gain | 0.235 |
| 3 | 129808799 | 129914958 | 106160 | *ALG1L2,FAM86HP* | 27 | gain | <0.001 |
| 3 | 149037585 | 149293316 | 255732 | *TM4SF18,TM4SF1,TM4SF4,WWTR1,U6* | 1 | gain | 0.235 |
| 3 | 188069330 | 188073872 | 4543 | *LPP* | 1 | gain | 0.235 |
| 3 | 194235453 | 194238390 | 2938 | *LINC00884* | 1 | gain | 0.235 |
| 3 | 195553259 | 195701151 | 147893 | *TNK2,AK127609,SDHAP1* | 1 | gain | 0.235 |
| 3 | 4035291 | 4042083 | 6793 | *SUMF1* | 1 | loss | 0.235 |
| 3 | 7496220 | 7616175 | 119956 | *GRM7* | 1 | loss | 0.235 |
| 3 | 23307096 | 23461395 | 154300 | *UBE2E2,MIR548AC* | 1 | loss | 0.235 |
| 3 | 52596398 | 52681122 | 84725 | *PBRM1* | 1 | loss | 0.235 |
| 3 | 52950391 | 53022855 | 72465 | *SFMBT1* | 1 | loss | 0.235 |
| 3 | 76073623 | 76097994 | 24372 | *ROBO2* | 1 | loss | 0.235 |
| 3 | 89419369 | 89419909 | 541 | *EPHA3* | 1 | loss | 0.235 |
| 3 | 94681412 | 94980130 | 298719 | *LINC00879* | 1 | loss | 0.235 |
| 3 | 99689368 | 99758465 | 69098 | *MIR548G,CMSS1,FILIP1L* | 1 | loss | 0.235 |
| 3 | 114499741 | 114545589 | 45849 | *ZBTB20* | 1 | loss | 0.235 |
| 3 | 122918197 | 122993710 | 75514 | *SEC22A* | 1 | loss | 0.235 |
| 3 | 129806912 | 129808799 | 1888 | *ALG1L2* | 1 | loss | 0.235 |
| 3 | 162487669 | 162512645 | 24977 | *BC073807* | 3 | loss | 0.013 |
| 3 | 162625983 | 162628602 | 2620 | *BC073807* | 1 | loss | 0.235 |
| 3 | 175222087 | 175243723 | 21637 | *NAALADL2* | 1 | loss | 0.235 |
| 4 | 66707 | 94380 | 27674 | *ZNF595,ZNF718* | 1 | gain | 0.235 |
| 4 | 3609390 | 3715650 | 106261 | *LOC100133461* | 1 | gain | 0.235 |
| 4 | 3885796 | 4190560 | 304765 | *DQ584669,FAM86EP,BC042823,OTOP1* | 14 | gain | <0.001 |
| 4 | 7570358 | 7591066 | 20709 | *SORCS2* | 1 | gain | 0.235 |
| 4 | 9486075 | 9681289 | 195215 | *MIR548I2,AB059369* | 8 | gain | <0.001 |
| 4 | 15638800 | 15991015 | 352216 | *FBXL5,FAM200B,BC017971,BST1,CD38,FGFBP1,FGFBP2,PROM1* | 2 | gain | 0.055 |
| 4 | 44009201 | 44586716 | 577516 | *KCTD8* | 1 | gain | 0.235 |
| 4 | 55093677 | 55120684 | 27008 | *PDGFRA* | 1 | gain | 0.235 |
| 4 | 58189742 | 59060539 | 870798 | *BC034799* | 1 | gain | 0.235 |
| 4 | 59907023 | 62728124 | 2821102 | *LPHN3* | 2 | gain | 0.055 |
| 4 | 76885216 | 76930902 | 45687 | *SDAD1,CXCL9* | 1 | gain | 0.235 |
| 4 | 116034368 | 116328595 | 294228 | *NDST4* | 1 | gain | 0.235 |
| 4 | 125403014 | 125481787 | 78774 | *AK057455* | 1 | gain | 0.235 |
| 4 | 132588909 | 132773067 | 184159 | *BC131768* | 1 | gain | 0.235 |
| 4 | 151174063 | 151183859 | 9797 | *DCLK2* | 1 | gain | 0.235 |
| 4 | 189258765 | 189404378 | 145614 | *LOC401164* | 1 | gain | 0.235 |
| 4 | 2146436 | 2155017 | 8582 | *POLN* | 1 | loss | 0.235 |
| 4 | 2196931 | 2237936 | 41006 | *POLN,HAUS3* | 1 | loss | 0.235 |
| 4 | 9501382 | 9744591 | 243210 | *MIR548I2,AB059369,DQ584669* | 1 | loss | 0.235 |
| 4 | 21640185 | 21992224 | 352040 | *KCNIP4,KCNIP4-IT1* | 1 | loss | 0.235 |
| 4 | 69371991 | 69410212 | 38222 | *UGT2B17* | 5 | loss | 0.001 |
| 4 | 71227212 | 71227802 | 591 | *SMR3A,SMR3B* | 1 | loss | 0.235 |
| 4 | 81949450 | 82116822 | 167373 | *BMP3,PRKG2* | 1 | loss | 0.235 |
| 4 | 102573942 | 102656858 | 82917 | *BANK1* | 1 | loss | 0.235 |
| 4 | 122285135 | 122292143 | 7009 | *QRFPR* | 1 | loss | 0.235 |
| 4 | 146812877 | 148129943 | 1317067 | *ZNF827,Y_RNA,LOC100505545,Mir_548,LSM6,SLC10A7,POU4F2,TTC29* | 1 | loss | 0.235 |
| 4 | 186260812 | 186309757 | 48946 | *SNX25,BC128459,LRP2BP* | 1 | loss | 0.235 |
| 5 | 125431 | 183401 | 57971 | *PLEKHG4B* | 1 | gain | 0.235 |
| 5 | 1749215 | 2197203 | 447989 | *MRPL36,NDUFS6,IRX4* | 1 | gain | 0.235 |
| 5 | 8383302 | 8495442 | 112141 | *LOC729506,LOC100505738,MIR4458* | 1 | gain | 0.235 |
| 5 | 32081411 | 32101168 | 19758 | *PDZD2* | 1 | gain | 0.235 |
| 5 | 35023034 | 35073760 | 50727 | *AGXT2,Mir_548,PRLR* | 1 | gain | 0.235 |
| 5 | 45589446 | 46334867 | 745422 | *HCN1* | 2 | gain | 0.055 |
| 5 | 68867282 | 70391241 | 1523960 | *GTF2H2C,AX748379,SMA,GUSBP3,LOC100272216,DQ574682,DQ596042,DQ571461,DQ575504,AK124130,SMA4,DQ570150,DQ591060,SERF1A,SERF1,SMN1,SMN2,BC045789,NAIP,DQ570835,SMA5,DQ587763,GTF2H2B,SMA3,LOC441081,GUSBP9,GTF2H2,LOC647859* | 1 | gain | 0.235 |
| 5 | 87262757 | 87507137 | 244381 | *TMEM161B* | 1 | gain | 0.235 |
| 5 | 112516709 | 112538755 | 22047 | *MCC* | 1 | gain | 0.235 |
| 5 | 172571590 | 172612565 | 40976 | *BNIP1* | 1 | gain | 0.235 |
| 5 | 180193767 | 180374484 | 180718 | *MGAT1,HEIH,LINC00847,ZFP62,BTNL8* | 3 | gain | 0.013 |
| 5 | 180433264 | 180527309 | 94046 | *BTNL3,BTNL9,TRNA_Val,TRNA_Leu* | 2 | gain | 0.055 |
| 5 | 54324190 | 54422714 | 98525 | *GZMK,Y_RNA,GZMA,CDC20B* | 1 | loss | 0.235 |
| 5 | 98646280 | 99150534 | 504255 | *DQ597441,DQ596041,DQ591060* | 1 | loss | 0.235 |
| 5 | 99590842 | 99849643 | 258802 | *LOC100133050,DQ574682,DQ596042,DQ583509* | 2 | loss | 0.055 |
| 5 | 107476634 | 107571304 | 94671 | *FBXL17* | 1 | loss | 0.235 |
| 5 | 109868874 | 109989103 | 120230 | *TMEM232* | 1 | loss | 0.235 |
| 5 | 112696567 | 112746390 | 49824 | *MCC,FLJ43978* | 1 | loss | 0.235 |
| 5 | 115409141 | 115426468 | 17328 | *COMMD10* | 1 | loss | 0.235 |
| 5 | 115489694 | 115493613 | 3920 | *COMMD10* | 1 | loss | 0.235 |
| 5 | 115534936 | 115537498 | 2563 | *COMMD10* | 1 | loss | 0.235 |
| 5 | 144772150 | 146559069 | 1786920 | *PRELID2,GRXCR2,SH3RF2,PLAC8L1,Mir_584,LARS,RBM27,POU4F3,TCERG1,GPR151,PPP2R2B,PPP2R2B-IT1* | 1 | loss | 0.235 |
| 5 | 178483445 | 178519187 | 35743 | *ZNF354C* | 1 | loss | 0.235 |
| 6 | 58082084 | 58774715 | 692632 | *TRNA_Ala,TRNA_Ile,TRNA_Met,GUSBP4,DQ596041,DQ587763,DQ596042,Mir_598* | 1 | gain | 0.235 |
| 6 | 66308556 | 66332540 | 23985 | *EYS* | 1 | gain | 0.235 |
| 6 | 111369737 | 111738004 | 368268 | *SLC16A10,KIAA1919,REV3L* | 1 | gain | 0.235 |
| 6 | 162590018 | 162680306 | 90289 | *PARK2* | 1 | gain | 0.235 |
| 6 | 162807682 | 162840211 | 32530 | *PARK2* | 1 | gain | 0.235 |
| 6 | 29836741 | 29837188 | 448 | *HLA-H,HLA-G* | 1 | loss | 0.235 |
| 6 | 31262461 | 31269522 | 7062 | *HLA-B* | 1 | loss | 0.235 |
| 6 | 31457633 | 31474688 | 17056 | *MICB* | 2 | loss | 0.055 |
| 6 | 32518757 | 32530286 | 11530 | *HLA-DRB5,HLA-DRB1,HLA-DRB6* | 1 | loss | 0.235 |
| 6 | 140455034 | 140571130 | 116097 | *5S_rRNA,MIR3668* | 1 | loss | 0.235 |
| 6 | 162187125 | 162402914 | 215790 | *PARK2* | 1 | loss | 0.235 |
| 6 | 162451920 | 162507690 | 55771 | *PARK2* | 2 | loss | 0.055 |
| 7 | 1203841 | 1638496 | 434656 | *AK090593,UNCX,MICALL2,AK127339,INTS1,MAFK,FW339998,TMEM184A,PSMG3,PSMG3-AS1,KIAA1908* | 1 | gain | 0.235 |
| 7 | 1806591 | 1962163 | 155573 | *MAD1L1,MIR4655,AK127048* | 1 | gain | 0.235 |
| 7 | 2808026 | 2947216 | 139191 | *GNA12,CARD11* | 1 | gain | 0.235 |
| 7 | 2961374 | 3317999 | 356626 | *CARD11,BC038729* | 1 | gain | 0.235 |
| 7 | 12596616 | 12619740 | 23125 | *BC075797,SCIN* | 1 | gain | 0.235 |
| 7 | 39058577 | 39062970 | 4394 | *POU6F2* | 1 | gain | 0.235 |
| 7 | 39137061 | 39545773 | 408713 | *POU6F2,Mir_548,POU6F2-AS1* | 1 | gain | 0.235 |
| 7 | 57120697 | 57377065 | 256369 | *ZNF479,GUSBP10,TRNA,MtDNA_ssA,TRNA_Pseudo* | 3 | gain | 0.013 |
| 7 | 65414064 | 65457437 | 43374 | *VKORC1L1,DQ594001,GUSB* | 2 | gain | 0.055 |
| 7 | 72425467 | 72762721 | 337255 | *NSUN5P2,TRIM74,BC073780,STAG3L3,LOC541473,FKBP6,PMS2L2,SPDYE8P,DQ596866,DQ586658,BC047594,BC110795,LOC100093631,NCF1B,NCF1,GTF2IRD2P1,BC018166,AK127026,NSUN5,TRIM50* | 1 | gain | 0.235 |
| 7 | 74102947 | 74142092 | 39146 | *GTF2I,BC070376* | 1 | gain | 0.235 |
| 7 | 83458529 | 83594123 | 135595 | *SEMA3A* | 1 | gain | 0.235 |
| 7 | 84744344 | 84821898 | 77555 | *SEMA3D* | 1 | gain | 0.235 |
| 7 | 93378717 | 93404813 | 26097 | *GNGT1* | 1 | gain | 0.235 |
| 7 | 97855578 | 97889672 | 34095 | *TECPR1* | 1 | gain | 0.235 |
| 7 | 104676522 | 105099343 | 422822 | *KMT2E,AF520793,AF520792,SRPK2,PUS7* | 1 | gain | 0.235 |
| 7 | 111666406 | 111713227 | 46822 | *DOCK4* | 1 | gain | 0.235 |
| 7 | 111911553 | 112031355 | 119803 | *ZNF277* | 1 | gain | 0.235 |
| 7 | 116365096 | 116443274 | 78179 | *MET,U7* | 1 | gain | 0.235 |
| 7 | 119313222 | 120144596 | 831375 | *KCND2* | 1 | gain | 0.235 |
| 7 | 127266882 | 127670004 | 403123 | *SND1,Mir_544,SND1-IT1,LRRC4* | 1 | gain | 0.235 |
| 7 | 133785365 | 133920443 | 135079 | *LRGUK* | 3 | gain | 0.013 |
| 7 | 143211643 | 143218396 | 6754 | *EPHA1-AS1* | 3 | gain | 0.013 |
| 7 | 145064742 | 145950454 | 885713 | *DQ597485,CNTNAP2* | 1 | gain | 0.235 |
| 7 | 146781980 | 146848581 | 66602 | *CNTNAP2* | 1 | gain | 0.235 |
| 7 | 151816202 | 151820821 | 4620 | *GALNT11* | 1 | gain | 0.235 |
| 7 | 158127608 | 158164002 | 36395 | *PTPRN2* | 1 | gain | 0.235 |
| 7 | 3509626 | 3618349 | 108724 | *SDK1,DL490859* | 1 | loss | 0.235 |
| 7 | 11369405 | 11374694 | 5290 | *BC040327* | 1 | loss | 0.235 |
| 7 | 11599456 | 11624109 | 24654 | *THSD7A* | 1 | loss | 0.235 |
| 7 | 16346544 | 16348763 | 2220 | *ISPD* | 1 | loss | 0.235 |
| 7 | 30809097 | 31011485 | 202389 | *INMT-FAM188B,FAM188B,AQP1,GHRHR* | 1 | loss | 0.235 |
| 7 | 57508724 | 57545549 | 36826 | *ZNF716* | 2 | loss | 0.055 |
| 7 | 110775577 | 110919924 | 144348 | *IMMP2L* | 1 | loss | 0.235 |
| 7 | 149372380 | 149481676 | 109297 | *TRNA_Cys,KRBA1,ZNF467,SSPO* | 1 | loss | 0.235 |
| 8 | 720491 | 1084728 | 364238 | *ERICH1-AS1* | 1 | gain | 0.235 |
| 8 | 2799369 | 3890012 | 1090644 | *CSMD1* | 1 | gain | 0.235 |
| 8 | 8087144 | 8102819 | 15676 | *FAM86B3P* | 2 | gain | 0.055 |
| 8 | 11881847 | 11934660 | 52814 | *DEFB130* | 2 | gain | 0.055 |
| 8 | 12542721 | 12587390 | 44670 | *LONRF1,MIR3926-1,MIR3926-2* | 5 | gain | 0.001 |
| 8 | 17319881 | 17476704 | 156824 | *SLC7A2,PDGFRL* | 1 | gain | 0.235 |
| 8 | 27763129 | 27853359 | 90231 | *SCARA5* | 1 | gain | 0.235 |
| 8 | 35337650 | 35401379 | 63730 | *UNC5D* | 1 | gain | 0.235 |
| 8 | 43161920 | 43353474 | 191555 | *POTEA* | 2 | gain | 0.055 |
| 8 | 59347361 | 59392737 | 45377 | *UBXN2B* | 1 | gain | 0.235 |
| 8 | 75793077 | 76234219 | 441143 | *CRISPLD1,BC062758* | 1 | gain | 0.235 |
| 8 | 95556054 | 95561026 | 4973 | *KIAA1429* | 1 | gain | 0.235 |
| 8 | 118170411 | 118198889 | 28479 | *SLC30A8,Metazoa_SRP* | 1 | gain | 0.235 |
| 8 | 119588257 | 119589373 | 1117 | *SAMD12* | 1 | gain | 0.235 |
| 8 | 121376284 | 121447878 | 71595 | *COL14A1,MRPL13* | 1 | gain | 0.235 |
| 8 | 131583439 | 131830985 | 247547 | *ADCY8* | 1 | gain | 0.235 |
| 8 | 133925027 | 133962414 | 37388 | *TG* | 1 | gain | 0.235 |
| 8 | 143634092 | 143750072 | 115981 | *ARC,JRK,JH8* | 1 | gain | 0.235 |
| 8 | 146195143 | 146298143 | 103001 | *ZNF252P,TMED10P1,ZNF252P-AS1,C8orf33* | 3 | gain | 0.013 |
| 8 | 4301996 | 4322234 | 20239 | *CSMD1* | 1 | loss | 0.235 |
| 8 | 4523483 | 4546567 | 23085 | *CSMD1* | 1 | loss | 0.235 |
| 8 | 13277834 | 13600240 | 322407 | *DLC1,C8orf48* | 1 | loss | 0.235 |
| 8 | 14488771 | 14642903 | 154133 | *SGCZ* | 1 | loss | 0.235 |
| 8 | 21690406 | 21747634 | 57229 | *DOK2* | 1 | loss | 0.235 |
| 8 | 21827833 | 21864540 | 36708 | *XPO7* | 2 | loss | 0.055 |
| 8 | 27624528 | 27676579 | 52052 | *CCDC25,ESCO2,PBK* | 1 | loss | 0.235 |
| 8 | 41820491 | 41917440 | 96950 | *KAT6A* | 1 | loss | 0.235 |
| 8 | 57788069 | 57843435 | 55367 | *U6* | 1 | loss | 0.235 |
| 8 | 63352500 | 63403700 | 51201 | *NKAIN3* | 1 | loss | 0.235 |
| 8 | 92109637 | 92118995 | 9359 | *LRRC69* | 2 | loss | 0.055 |
| 8 | 133795608 | 133836098 | 40491 | *PHF20L1* | 1 | loss | 0.235 |
| 8 | 136620080 | 138711817 | 2091738 | *KHDRBS3,U1* | 2 | loss | 0.055 |
| 8 | 143956808 | 143994112 | 37305 | *CYP11B1,CYP11B2* | 2 | loss | 0.055 |
| 9 | 46587 | 224462 | 177876 | *AY343892,AY343902,FOXD4,CBWD1,C9orf66,DOCK8* | 3 | gain | 0.013 |
| 9 | 15272230 | 15274373 | 2144 | *TTC39B* | 1 | gain | 0.235 |
| 9 | 22190562 | 22988892 | 798331 | *DMRTA1,FLJ35282* | 1 | gain | 0.235 |
| 9 | 68176154 | 68683835 | 507682 | *AK308561,BC080605,LOC642236* | 17 | gain | <0.001 |
| 9 | 88663471 | 88709450 | 45980 | *GOLM1* | 1 | gain | 0.235 |
| 9 | 101342282 | 101674672 | 332391 | *GABBR2,ANKS6,GALNT12* | 1 | gain | 0.235 |
| 9 | 108348889 | 108398440 | 49552 | *FKTN* | 1 | gain | 0.235 |
| 9 | 125422424 | 125947085 | 524662 | *OR1L1,OR1L3,OR1L4,OR1L6,OR5C1,OR1K1,BC010186,PDCL,AL833455,RC3H2,SNORD90,ZBTB6,ZBTB26,RABGAP1,GPR21,MIR600HG,DQ582309,STRBP* | 1 | gain | 0.235 |
| 9 | 140994201 | 141091382 | 97182 | *CACNA1B,TUBBP5* | 1 | gain | 0.235 |
| 9 | 10145099 | 10176877 | 31779 | *PTPRD* | 1 | loss | 0.235 |
| 9 | 28813877 | 28893377 | 79501 | *LINGO2,MIR876,MIR873* | 1 | loss | 0.235 |
| 9 | 37048277 | 37092433 | 44157 | *LOC100506710* | 2 | loss | 0.055 |
| 9 | 71387063 | 71419664 | 32602 | *PIP5K1B,FAM122A* | 1 | loss | 0.235 |
| 9 | 105886214 | 105917090 | 30877 | *BC035187* | 1 | loss | 0.235 |
| 9 | 115735911 | 115820554 | 84644 | *ZNF883,ZFP37* | 1 | loss | 0.235 |
| 9 | 116314918 | 117370538 | 1055621 | *RGS3,Mir_584,ZNF618,AMBP,KIF12,COL27A1,MIR455,ORM1,ORM2,AKNA,AX746484,DFNB31,ATP6V1G1* | 1 | loss | 0.235 |
| 9 | 132611341 | 132613251 | 1911 | *USP20* | 1 | loss | 0.235 |
| 9 | 135172412 | 135227676 | 55265 | *SETX* | 1 | loss | 0.235 |
| 10 | 1708625 | 1780944 | 72320 | *ADARB2* | 1 | gain | 0.235 |
| 10 | 4962571 | 5063145 | 100575 | *AKR1C1,AKR1C3,AKR1C2* | 1 | gain | 0.235 |
| 10 | 11899207 | 11904073 | 4867 | *PROSER2,PROSER2-AS1* | 1 | gain | 0.235 |
| 10 | 34670528 | 35328422 | 657895 | *PARD3,CUL2* | 2 | gain | 0.055 |
| 10 | 38679109 | 39076233 | 397125 | *SEPT7P9,LOC399744,ACTR3BP5* | 1 | gain | 0.235 |
| 10 | 74893963 | 75007588 | 113626 | *ECD,FAM149B1,DNAJC9,MRPS16,DNAJC9-AS1* | 1 | gain | 0.235 |
| 10 | 81448995 | 81598043 | 149049 | *AX747158,DQ586890,LOC642361* | 1 | gain | 0.235 |
| 10 | 128138653 | 128388262 | 249610 | *C10orf90* | 1 | gain | 0.235 |
| 10 | 5736419 | 5827074 | 90656 | *FAM208B,C10orf18,GDI2* | 1 | loss | 0.235 |
| 10 | 29594107 | 29617150 | 23044 | *LYZL1* | 1 | loss | 0.235 |
| 10 | 46996235 | 47000587 | 4353 | *GPRIN2* | 1 | loss | 0.235 |
| 10 | 48883390 | 49322869 | 439480 | *AGAP8,DQ588224,BMS1P1,GLUD1P7,FAM25C,LOC399753,AK309922* | 2 | loss | 0.055 |
| 10 | 49569239 | 49627912 | 58674 | *MAPK8* | 1 | loss | 0.235 |
| 10 | 65048713 | 65091573 | 42861 | *JMJD1C* | 1 | loss | 0.235 |
| 10 | 68024502 | 68109326 | 84825 | *CTNNA3* | 2 | loss | 0.055 |
| 10 | 88206702 | 88278582 | 71881 | *WAPAL* | 1 | loss | 0.235 |
| 10 | 119072166 | 119108971 | 36806 | *PDZD8* | 1 | loss | 0.235 |
| 11 | 2936846 | 2986776 | 49931 | *SLC22A18,PHLDA2,NAP1L4,SNORA54* | 1 | gain | 0.235 |
| 11 | 3421731 | 3624237 | 202507 | *LOC650368* | 2 | gain | 0.055 |
| 11 | 4359044 | 4391529 | 32486 | *OR52B4* | 2 | gain | 0.055 |
| 11 | 5487596 | 5497617 | 10022 | *HBE1,OR51B5,HBG2* | 1 | gain | 0.235 |
| 11 | 5935498 | 5944221 | 8724 | *TRIM5* | 1 | gain | 0.235 |
| 11 | 12505324 | 12571873 | 66550 | *PARVA* | 1 | gain | 0.235 |
| 11 | 21908469 | 22242623 | 334155 | *ANO5* | 1 | gain | 0.235 |
| 11 | 36062969 | 36294034 | 231066 | *BC036209,LDLRAD3,COMMD9* | 1 | gain | 0.235 |
| 11 | 49821330 | 49853627 | 32298 | *LOC440040* | 1 | gain | 0.235 |
| 11 | 76813595 | 76888643 | 75049 | *OMP,CAPN5,MYO7A* | 1 | gain | 0.235 |
| 11 | 83559542 | 83594651 | 35110 | *DLG2* | 1 | gain | 0.235 |
| 11 | 86182720 | 86323285 | 140566 | *ME3* | 1 | gain | 0.235 |
| 11 | 129532342 | 129663037 | 130696 | *AX746800* | 1 | gain | 0.235 |
| 11 | 134752371 | 134891347 | 138977 | *AK130852* | 1 | gain | 0.235 |
| 11 | 2614726 | 2664528 | 49803 | *KCNQ1OT1,KCNQ1* | 1 | loss | 0.235 |
| 11 | 5492033 | 5493384 | 1352 | *HBE1,OR51B5,HBG2* | 1 | loss | 0.235 |
| 11 | 61387662 | 61419944 | 32283 | *RPLP0P2* | 1 | loss | 0.235 |
| 11 | 66918617 | 66960393 | 41777 | *KDM2A* | 1 | loss | 0.235 |
| 11 | 81749175 | 81978632 | 229458 | *BC041900* | 1 | loss | 0.235 |
| 11 | 88373718 | 88374069 | 352 | *GRM5* | 1 | loss | 0.235 |
| 11 | 89444427 | 89659556 | 215130 | *TRIM77,TRIM49,LOC642414,TRIM53AP,TRIM64B,TRIM49D2P* | 1 | loss | 0.235 |
| 11 | 120238962 | 120357547 | 118586 | *ARHGEF12* | 1 | loss | 0.235 |
| 11 | 124871538 | 124923107 | 51570 | *CCDC15* | 1 | loss | 0.235 |
| 11 | 134152030 | 134210850 | 58821 | *GLB1L3,GLB1L2* | 1 | loss | 0.235 |
| 11 | 134355897 | 134472198 | 116302 | *LOC283177,AK095081* | 1 | loss | 0.235 |
| 12 | 591300 | 635259 | 43960 | *B4GALNT3* | 1 | gain | 0.235 |
| 12 | 7865017 | 7878255 | 13239 | *DPPA3* | 1 | gain | 0.235 |
| 12 | 8378239 | 8536600 | 158362 | *FAM90A1,FAM86FP,LINC00937* | 5 | gain | 0.001 |
| 12 | 19368176 | 19431725 | 63550 | *PLEKHA5* | 1 | gain | 0.235 |
| 12 | 48903304 | 48949043 | 45740 | *OR8S1* | 1 | gain | 0.235 |
| 12 | 59031732 | 59262663 | 230932 | *AK093124* | 1 | gain | 0.235 |
| 12 | 100702496 | 100758471 | 55976 | *SCYL2,SLC17A8* | 1 | gain | 0.235 |
| 12 | 102294618 | 102359923 | 65306 | *DRAM1* | 1 | gain | 0.235 |
| 12 | 129874776 | 129886386 | 11611 | *TMEM132D* | 1 | gain | 0.235 |
| 12 | 130579093 | 131130277 | 551185 | *FZD10-AS1,FZD10,PIWIL1,RIMBP2* | 1 | gain | 0.235 |
| 12 | 150430 | 203640 | 53211 | *IQSEC3* | 1 | loss | 0.235 |
| 12 | 1788033 | 1878392 | 90360 | *ADIPOR2* | 2 | loss | 0.055 |
| 12 | 42481443 | 42504320 | 22878 | *GXYLT1* | 1 | loss | 0.235 |
| 12 | 47998378 | 48093272 | 94895 | *RPAP3* | 1 | loss | 0.235 |
| 13 | 19395626 | 19418876 | 23251 | *ANKRD20A9P* | 1 | gain | 0.235 |
| 13 | 20545714 | 20654610 | 108897 | *ZMYM2,BC044596* | 1 | gain | 0.235 |
| 13 | 43463299 | 43750413 | 287115 | *EPSTI1,DNAJC15* | 1 | gain | 0.235 |
| 13 | 85980783 | 86025976 | 45194 | *LINC00351* | 1 | gain | 0.235 |
| 13 | 95513006 | 95565992 | 52987 | *BC045767* | 1 | gain | 0.235 |
| 13 | 95920458 | 95926431 | 5974 | *ABCC4* | 2 | gain | 0.055 |
| 13 | 115064635 | 115108385 | 43751 | *UPF3A,CHAMP1* | 1 | gain | 0.235 |
| 13 | 25072527 | 25133346 | 60820 | *PARP4* | 2 | loss | 0.055 |
| 13 | 35922932 | 36164985 | 242054 | *NBEA,MIR548F5* | 1 | loss | 0.235 |
| 13 | 67437089 | 67590005 | 152917 | *PCDH9,PCDH9-AS2,PCDH9-AS3* | 1 | loss | 0.235 |
| 13 | 93039812 | 93045491 | 5680 | *GPC5* | 1 | loss | 0.235 |
| 13 | 95387281 | 95505262 | 117982 | *BC045767* | 1 | loss | 0.235 |
| 13 | 111369656 | 111406396 | 36741 | *ING1* | 1 | loss | 0.235 |
| 13 | 114987446 | 115108385 | 120940 | *CDC16,UPF3A,CHAMP1* | 1 | loss | 0.235 |
| 14 | 24500989 | 24513905 | 12917 | *DHRS4L2,DHRS4L1* | 1 | gain | 0.235 |
| 14 | 63352686 | 63408481 | 55796 | *KCNH5* | 1 | gain | 0.235 |
| 14 | 67815213 | 67819602 | 4390 | *ATP6V1D* | 1 | gain | 0.235 |
| 14 | 74030719 | 74076979 | 46261 | *ACOT1,ACOT2,TRNA_Pseudo,ACOT4* | 1 | gain | 0.235 |
| 14 | 78391384 | 78452114 | 60731 | *ADCK1* | 1 | gain | 0.235 |
| 14 | 78633762 | 78697857 | 64096 | *5S_rRNA,NRXN3* | 1 | gain | 0.235 |
| 14 | 92342442 | 92394310 | 51869 | *FBLN5* | 1 | gain | 0.235 |
| 14 | 101489161 | 101525989 | 36829 | *MIR411,MIR299,Mir_154,MIR380,MIR1197,MIR323A,MIR758,MIR329-1,MIR329-2,MIR494,MIR1193,MIR543,MIR495,JA715142,MIR376C,Mir_654,MIR376A2,MIR654,MIR376B,MIR376A1,MIR300,MIR1185-1,MIR1185-2,MIR381,MIR487B,MIR539,MIR889,Mir_544,MIR655,MIR487A,MIR382,MIR134,MIR668,MIR485,MIR323B* | 1 | gain | 0.235 |
| 14 | 24429298 | 24444817 | 15520 | *DHRS4,DHRS4L2,DHRS4L1* | 3 | loss | 0.013 |
| 14 | 24500633 | 24500989 | 357 | *DHRS4L2,DHRS4L1* | 1 | loss | 0.235 |
| 14 | 75244826 | 75290933 | 46108 | *YLPM1* | 1 | loss | 0.235 |
| 14 | 94644588 | 94741119 | 96532 | *PPP4R4* | 1 | loss | 0.235 |
| 14 | 99861751 | 99950570 | 88820 | *SETD3,CCNK* | 1 | loss | 0.235 |
| 14 | 101381713 | 101464984 | 83272 | *SNORD112,SNORD113-1,SNORD113-2,SNORD113,SNORD113-4,SNORD113-5,SNORD113-6,SNORD113-7,SNORD113-9,SNORD114-1,SNORD114-2,SNORD114-3,SNORD114-4,SNORD114-5,SNORD114-6,SNORD114-7,SNORD114-8,SNORD114-9,SNORD114-10,SNORD114-11,SNORD114-12,SNORD114-13,SNORD114-14,SNORD114-15,SNORD114-16,SNORD114-17,SNORD114-18,SNORD114-19,SNORD114-20,SNORD114-21,SNORD114-22,SNORD114-23,SNORD114-24,SNORD114-25,SNORD114-26,SNORD114-27,SNORD114-28,SNORD114-29,SNORD114-30,SNORD114-31* | 1 | loss | 0.235 |
| 15 | 28709280 | 29073541 | 364262 | *MIR4509-1,GOLGA8G,JB175342,DQ578700,GOLGA8F,DQ588687,DQ599733,GOLGA6L1,DQ600136,DQ579907,LOC283767,DQ582071,AK311660,AK309255,AK307870,DQ593342,HERC2P9,DQ596685,DQ582448,DQ593032,DQ582939,DQ597560,WHAMMP2,LOC100289656,LOC646278* | 2 | gain | 0.055 |
| 15 | 33145711 | 33546098 | 400388 | *FMN1,SNORD77,TMCO5B* | 1 | gain | 0.235 |
| 15 | 44579854 | 44811366 | 231513 | *CASC4,CTDSPL2* | 1 | gain | 0.235 |
| 15 | 59744010 | 60031884 | 287875 | *FAM81A,GCNT3,GTF2A2,BNIP2* | 1 | gain | 0.235 |
| 15 | 71669115 | 71672753 | 3639 | *THSD4* | 1 | gain | 0.235 |
| 15 | 84915080 | 85089609 | 174530 | *DQ586822,DQ582071,DQ578199,DQ574758,DQ576060,DQ581594,DQ601279,DNM1P41,GOLGA6L5,DQ582073,UBE2Q2P1* | 1 | gain | 0.235 |
| 15 | 102270164 | 102317809 | 47646 | *BC101079,DQ597539,DQ593624,DQ593864,DQ582666,DQ576888,DQ575740,DQ582460,DQ593630,DQ596486,DQ582294,DQ595661,DQ588439,DQ593353,DQ597703,DQ585237,DQ588452,DQ593627,DQ586526,DQ588428,DQ571896,DQ588425,DQ588362,DQ578285,DQ597461,DQ586138,DQ578289,DQ583497,DQ576933,DQ600537,DQ573820,DQ576896,DQ586246,DQ579816,DQ595216,DQ582610,DQ588066,DQ576999,DQ588584,DQ591415,DQ585242,DQ582462,DQ576947,DQ588394,DQ588329,DQ588370,DQ588406,DQ587115,DQ601306,DQ598070,DQ588424,DQ597025,DQ588388,DQ573799,DQ590394,DQ574757,DQ599785,DQ575242,DQ586415,DQ592653,DQ583348,DQ577251,DQ593367,DQ592190,DQ588143,DQ596604,DQ578010,DQ599787,DQ588124,DQ572823,DQ580168,DQ601694,DQ582073,DQ571638,DQ570882,DQ593032,DQ589204,DQ578258,DQ571326,DQ576544* | 1 | gain | 0.235 |
| 15 | 70936438 | 70992543 | 56106 | *UACA* | 1 | loss | 0.235 |
| 15 | 86794435 | 86813735 | 19301 | *AGBL1* | 1 | loss | 0.235 |
| 15 | 89791516 | 89847735 | 56220 | *FANCI* | 1 | loss | 0.235 |
| 15 | 94843381 | 94863837 | 20457 | *MCTP2* | 1 | loss | 0.235 |
| 16 | 60765 | 110858 | 50094 | *DDX11L10,WASH1,BC032901,POLR3K,SNRNP25,RHBDF1* | 1 | gain | 0.235 |
| 16 | 5942659 | 7000800 | 1058142 | *U7,RBFOX1* | 1 | gain | 0.235 |
| 16 | 8727484 | 8782971 | 55488 | *METTL22,BX537921,ABAT* | 1 | gain | 0.235 |
| 16 | 8853060 | 8997244 | 144185 | *ABAT,TMEM186,PMM2,CARHSP1,USP7* | 2 | gain | 0.055 |
| 16 | 29591757 | 30191895 | 600139 | *LOC440354,SLC7A5P1,SPN,QPRT,C16orf54,BC041466,ZG16,KIF22,MAZ,AB209061,AK097472,PRRT2,BC029255,PAGR1,MVP,CDIPT,CDIPT-AS1,SEZ6L2,ASPHD1,KCTD13,TMEM219,TAOK2,HIRIP3,INO80E,DOC2A,C16orf92,FAM57B,AK097527,ALDOA,PPP4C,TBX6,YPEL3,AK097453,GDPD3,MAPK3,BOLA2* | 1 | gain | 0.235 |
| 16 | 33864347 | 34011271 | 146925 | *JB158072,LINC00273,JB175072* | 2 | gain | 0.055 |
| 16 | 34197339 | 34503222 | 305884 | *UBE2MP1* | 1 | gain | 0.235 |
| 16 | 55895285 | 55985580 | 90296 | *CES5A* | 1 | gain | 0.235 |
| 16 | 5010913 | 5216718 | 205806 | *SEC14L5,NAGPA,NAGPA-AS1,C16orf89,ALG1,FAM86A* | 1 | loss | 0.235 |
| 16 | 5632426 | 5785405 | 152980 | *BC108660* | 1 | loss | 0.235 |
| 16 | 6425429 | 6505456 | 80028 | *RBFOX1* | 1 | loss | 0.235 |
| 16 | 12700163 | 12756860 | 56698 | *CPPED1* | 1 | loss | 0.235 |
| 16 | 16290242 | 16330478 | 40237 | *ABCC6,NOMO3* | 1 | loss | 0.235 |
| 16 | 31560514 | 31656578 | 96065 | *YBX3P1* | 1 | loss | 0.235 |
| 16 | 74647795 | 74707037 | 59243 | *RFWD3,MLKL* | 1 | loss | 0.235 |
| 16 | 76850155 | 76905308 | 55154 | *MIR4719* | 1 | loss | 0.235 |
| 16 | 78405766 | 78426104 | 20339 | *WWOX* | 2 | loss | 0.055 |
| 16 | 78807770 | 78847523 | 39754 | *WWOX* | 1 | loss | 0.235 |
| 16 | 83042326 | 83048807 | 6482 | *CDH13* | 1 | loss | 0.235 |
| 17 | 575792 | 577890 | 2099 | *VPS53* | 1 | gain | 0.235 |
| 17 | 3157892 | 3190846 | 32955 | *OR3A2* | 1 | gain | 0.235 |
| 17 | 8282211 | 8320276 | 38066 | *RPL26,RNF222* | 1 | gain | 0.235 |
| 17 | 13410561 | 13463086 | 52526 | *HS3ST3A1* | 1 | gain | 0.235 |
| 17 | 18496294 | 18793913 | 297620 | *CCDC144B,TBC1D28,ZNF286B,FOXO3B,TRIM16L,FBXW10,TVP23B,PRPSAP2* | 1 | gain | 0.235 |
| 17 | 25425374 | 25639651 | 214278 | *MIR4522,WSB1* | 1 | gain | 0.235 |
| 17 | 54632489 | 54673529 | 41041 | *NOG* | 1 | gain | 0.235 |
| 17 | 78103013 | 78143441 | 40429 | *EIF4A3* | 1 | gain | 0.235 |
| 17 | 78223817 | 78317442 | 93626 | *SLC26A11,RNF213* | 1 | gain | 0.235 |
| 17 | 78951153 | 79505624 | 554472 | *CHMP6,AF258550,BAIAP2-AS1,BAIAP2,AATK,MIR657,MIR3065,MIR338,MIR1250,AATK-AS1,AZI1,ENTHD2,AL832593,C17orf89,SLC38A10,LINC00482,TMEM105,LOC100130370,BAHCC1,MIR4740,MIR3186,ACTG1,DQ585569,FSCN2* | 1 | gain | 0.235 |
| 17 | 79619226 | 80178991 | 559766 | *PDE6G,OXLD1,CCDC137,ARL16,HGS,MRPL12,SLC25A10,GCGR,FAM195B,PPP1R27,P4HB,AK293147,ARHGDIA,ALYREF,ANAPC11,NPB,PCYT2,SIRT7,MAFG,MAFG-AS1,PYCR1,MYADML2,NOTUM,ASPSCR1,STRA13,LRRC45,RAC3,DCXR,RFNG,BC050399,GPS1,DUS1L,FASN,CCDC57* | 1 | gain | 0.235 |
| 17 | 833790 | 1516480 | 682691 | *NXN,TIMM22,ABR,MIR3183,Metazoa_SRP,BHLHA9,TUSC5,YWHAE,CRK,MYO1C,INPP5K,PITPNA-AS1,PITPNA,SLC43A2* | 1 | loss | 0.235 |
| 17 | 21347937 | 21524160 | 176224 | *C17orf51* | 1 | loss | 0.235 |
| 17 | 33684107 | 33766206 | 82100 | *SLFN11,SLFN12,SLFN13* | 1 | loss | 0.235 |
| 17 | 37989037 | 38041762 | 52726 | *IKZF3,ZPBP2* | 1 | loss | 0.235 |
| 17 | 44135795 | 44198145 | 62351 | *KANSL1* | 1 | loss | 0.235 |
| 17 | 51032228 | 51067847 | 35620 | *C17orf112* | 1 | loss | 0.235 |
| 17 | 64408643 | 64486268 | 77626 | *PRKCA,BC033554* | 1 | loss | 0.235 |
| 18 | 543161 | 1855370 | 1312210 | *CETN1,CLUL1,C18orf56,TYMS,ENOSF1,YES1,ADCYAP1,LINC00470,7SK* | 1 | gain | 0.235 |
| 18 | 2850631 | 3149505 | 298875 | *EMILIN2,LPIN2,LOC727896,MYOM1* | 2 | gain | 0.235 |
| 18 | 4092326 | 4262436 | 170111 | *DLGAP1* | 1 | gain | 0.235 |
| 18 | 11991132 | 12146344 | 155213 | *IMPA2,ANKRD62* | 1 | gain | 0.235 |
| 18 | 35154734 | 35274823 | 120090 | *MIR4318* | 1 | gain | 0.235 |
| 18 | 44564162 | 44615322 | 51161 | *KATNAL2* | 1 | gain | 0.235 |
| 18 | 60347199 | 60724901 | 377703 | *PHLPP1* | 1 | gain | 0.235 |
| 18 | 12090696 | 12163129 | 72434 | *ANKRD62* | 1 | loss | 0.235 |
| 18 | 45346947 | 45404768 | 57822 | *SMAD2* | 1 | loss | 0.235 |
| 18 | 45437808 | 45445677 | 7870 | *SMAD2* | 1 | loss | 0.235 |
| 18 | 53140533 | 53435469 | 294937 | *TCF4,MIR4529* | 1 | loss | 0.235 |
| 18 | 76946129 | 77063910 | 117782 | *ATP9B* | 1 | loss | 0.235 |
| 19 | 4073716 | 4196014 | 122299 | *MAP2K2,CREB3L3,SIRT6,ANKRD24* | 1 | gain | 0.235 |
| 19 | 7112111 | 7212256 | 100146 | *INSR* | 1 | gain | 0.235 |
| 19 | 12516150 | 12546189 | 30040 | *ZNF443* | 1 | gain | 0.235 |
| 19 | 15676750 | 15730101 | 53352 | *CYP4F8* | 1 | gain | 0.235 |
| 19 | 22084265 | 22152043 | 67779 | *ZNF208* | 2 | gain | 0.055 |
| 19 | 23212272 | 23246400 | 34129 | *BC037873* | 1 | gain | 0.235 |
| 19 | 23491237 | 23532008 | 40772 | *BC038574* | 1 | gain | 0.235 |
| 19 | 36752663 | 36852733 | 100071 | *LOC100134317,LINC00665,AX746638,ZFP14* | 1 | gain | 0.235 |
| 19 | 36915031 | 36973918 | 58888 | *ZNF566* | 1 | gain | 0.235 |
| 19 | 39257977 | 39308190 | 50214 | *LGALS7,LGALS7B,LGALS4,ECH1,HNRNPL* | 1 | gain | 0.235 |
| 19 | 41451652 | 41519615 | 67964 | *CYP2A7,CYP2B7P1,CYP2B6* | 2 | gain | 0.055 |
| 19 | 43280216 | 43294378 | 14163 | *PSG3,PSG1* | 2 | gain | 0.055 |
| 19 | 47282844 | 47418388 | 135545 | *SLC1A5,SNAR-E,AP2S1* | 1 | gain | 0.235 |
| 19 | 49064771 | 49392718 | 327948 | *SULT2B1,FAM83E,SPACA4,RPL18,SPHK2,DBP,CA11,SEC1P,Mir_324,NTN5,FUT2,MAMSTR,RASIP1,IZUMO1,FUT1,FGF21,BCAT2,HSD17B14,PLEKHA4,PPP1R15A,TULP2* | 1 | gain | 0.235 |
| 19 | 53304209 | 53316328 | 12120 | *ZNF28* | 2 | gain | 0.055 |
| 19 | 53518514 | 53544965 | 26452 | *ERVV-1* | 1 | gain | 0.235 |
| 19 | 54223171 | 54265278 | 42108 | *MIR520D,MIR517B,MIR520G,MIR516B2,MIR526A2,MIR518E,MIR518A1,MIR518D,MIR516B1,MIR518A2,MIR517C,MIR520H,MIR521-1,MIR522,MIR519A1,MIR527,MIR516A1,MIR1283-2,MIR516A2* | 2 | gain | 0.055 |
| 19 | 54717958 | 54730202 | 12245 | *LILRB3,LILRA6* | 1 | gain | 0.235 |
| 19 | 54781843 | 54925091 | 143249 | *LILRA6,LILRB2,MIR4752,LILRA3,LILRA5,LILRA4,LAIR1* | 1 | gain | 0.235 |
| 19 | 55237234 | 55248107 | 10874 | *KIR3DL3* | 2 | gain | 0.055 |
| 19 | 55360538 | 56717229 | 1356692 | *KIR3DL1,KIR2DS4,KIR3DL2,FCAR,NCR1,NLRP7,RNU6-64P,NLRP2,GP6,RDH13,AL833150,AK122764,EPS8L1,PPP1R12C,Mir_324,TNNT1,TNNI3,DNAAF3,AK097618,SYT5,PTPRH,BC034929,TMEM86B,PPP6R1,HSPBP1,X05128,BRSK1,TMEM150B,SUV420H2,COX6B2,FAM71E2,IL11,TMEM190,TMEM238,RPL28,UBE2S,BC047644,SHISA7,ISOC2,ZNF628,NAT14,SSC5D,SBK2,SGK110,ZNF579,FIZ1,ZNF524,ZNF865,ZNF784,ZNF581,ZNF580,CCDC106,U2AF2,EPN1,NLRP9,RFPL4A,RFPL4AL1,NLRP11,NLRP4,NLRP13,NLRP8,NLRP5,ZNF787,ZNF444,GALP,ZSCAN5B* | 4 | gain | 0.003 |
| 19 | 56892868 | 57544489 | 651622 | *ZNF582,ZNF582-AS1,ZNF583,ZNF667,ZNF667-AS1,ZNF471,ZFP28,BX647249,ZNF470,ZNF71,SMIM17,ZNF835,FJ997633,BC036412,ZIM2,PEG3,PEG3-AS1,MIMT1* | 1 | gain | 0.235 |
| 19 | 57644611 | 58107311 | 462701 | *ZIM3,DUXA,ZNF264,AURKC,ZNF805,ZNF460,ZNF543,ZNF304,TRAPPC2P1,ZNF547,ZNF548,ZNF17,ZNF749,VN1R1,ZNF772,ZNF419,ZNF773,ZNF549,ZNF550,ZNF416,ZIK1* | 1 | gain | 0.235 |
| 19 | 58506058 | 59097842 | 591785 | *ZNF606,LOC100128398,ZSCAN1,ZNF135,ZSCAN18,ZNF329,ZNF274,ZNF544,BC063675,ZNF8,LOC113386,ZSCAN22,A1BG,A1BG-AS1,ZNF497,BC023201,ZNF837,MIR4754,RPS5,LOC646862,ZNF584,DQ581862,ZNF132,ZNF324B,ZNF324,ZNF446,SLC27A5,ZBTB45,TRIM28,CHMP2A,UBE2M,LOC100131691,MZF1,MGC2752* | 1 | gain | 0.235 |
| 19 | 30436408 | 30504073 | 67666 | *URI1* | 1 | loss | 0.235 |
| 19 | 52127030 | 52148185 | 21156 | *SIGLEC5,SIGLEC14* | 1 | loss | 0.235 |
| 19 | 53357173 | 53370013 | 12841 | *ZNF468,ZNF320* | 1 | loss | 0.235 |
| 19 | 53518747 | 53552217 | 33471 | *ERVV-1,ERVV-2* | 1 | loss | 0.235 |
| 19 | 54256959 | 54279108 | 22150 | *MIR527,MIR516A1,MIR1283-2,MIR516A2,MIR519A2* | 1 | loss | 0.235 |
| 19 | 55348691 | 55360538 | 11848 | *KIR2DS4,KIR3DL1* | 1 | loss | 0.235 |
| 19 | 57877521 | 57931028 | 53508 | *ZNF547,ZNF548,ZNF17* | 1 | loss | 0.235 |
| 20 | 1599142 | 1599572 | 431 | *SIRPB1* | 1 | gain | 0.235 |
| 20 | 8516056 | 8573282 | 57227 | *PLCB1* | 1 | gain | 0.235 |
| 20 | 25496702 | 25701264 | 204563 | *NINL,NANP,ZNF337* | 1 | gain | 0.235 |
| 20 | 35700633 | 35718567 | 17935 | *RBL1* | 1 | gain | 0.235 |
| 20 | 40117905 | 40405471 | 287567 | *Mir_147,CHD6,U6* | 1 | gain | 0.235 |
| 20 | 58224682 | 59007873 | 783192 | *PHACTR3,SYCP2,PPP1R3D,FAM217B,CDH26,C20orf197,LOC284757* | 2 | gain | 0.055 |
| 20 | 25810309 | 25871906 | 61598 | *BX648489,FAM182B* | 1 | loss | 0.235 |
| 20 | 29479828 | 29601767 | 121940 | *Y_RNA* | 1 | loss | 0.235 |
| 20 | 41234101 | 41240852 | 6752 | *PTPRT* | 2 | loss | 0.055 |
| 20 | 58423049 | 58513089 | 90041 | *SYCP2,PPP1R3D,FAM217B* | 1 | loss | 0.235 |
| 21 | 14484409 | 14594223 | 109815 | *ANKRD30BP2* | 8 | gain | <0.001 |
| 21 | 15255416 | 15285842 | 30427 | *DQ579288,DQ587539,DQ579969* | 2 | gain | 0.055 |
| 21 | 18880915 | 18899229 | 18315 | *CXADR* | 1 | gain | 0.235 |
| 21 | 19374322 | 19378086 | 3765 | *CHODL* | 1 | gain | 0.235 |
| 21 | 23916594 | 24738328 | 821735 | *D21S2088E* | 1 | gain | 0.235 |
| 21 | 43836186 | 43893628 | 57443 | *UBASH3A,U6,RSPH1* | 1 | gain | 0.235 |
| 21 | 48050388 | 48096945 | 46558 | *PRMT2* | 2 | gain | 0.055 |
| 21 | 10956140 | 11042834 | 86695 | *TPTE,BAGE3* | 1 | loss | 0.235 |
| 21 | 14456404 | 14594223 | 137820 | *ANKRD30BP2* | 1 | loss | 0.235 |
| 21 | 23358347 | 23388794 | 30448 | *BC039377* | 1 | loss | 0.235 |
| 21 | 40537673 | 40570740 | 33068 | *PSMG1,BRWD1* | 1 | loss | 0.235 |
| 21 | 44973667 | 45065167 | 91501 | *HSF2BP,DQ577420* | 1 | loss | 0.235 |
| 21 | 47067182 | 47209452 | 142271 | *PCBP3* | 1 | loss | 0.235 |
| 22 | 18640300 | 18672873 | 32574 | *USP18,AK129567* | 1 | gain | 0.235 |
| 22 | 19006984 | 19568989 | 562006 | *DGCR5,DGCR9,DGCR10,DGCR2,Y_RNA,DGCR11,DGCR14,TSSK2,GSC2,SLC25A1,CLTCL1,HIRA,MRPL40,C22orf39,BX648073,UFD1L,U84523,CDC45,CLDN5,LINC00895* | 1 | gain | 0.235 |
| 22 | 19573160 | 21442671 | 1869512 | *SEPT5-GP1BB,TBX1,GNB1L,C22orf29,TXNRD2,COMT,MIR4761,ARVCF,TANGO2,MIR185,DGCR8,MIR3618,MIR1306,TRMT2A,RANBP1,ZDHHC8,LOC388849,LOC284865,LINC00896,RTN4R,MIR1286,KIAA1653,DGCR6L,LOC729444,TMEM191B,PI4KAP1,HV593096,HV593134,HV593127,HV593178,HV593135,7SK,RIMBP3,Metazoa_SRP,AK129567,AK302545,JX456220,USP41,ZNF74,SCARF2,KLHL22,MED15,BC035867,POM121L4P,DQ571461,TMEM191A,PI4KA,HV593183,HV593110,SERPIND1,SNAP29,CRKL,BC033281,BC127858,AIFM3,LZTR1,THAP7,DQ574263,THAP7-AS1,TUBA3FP,P2RX6,SLC7A4,Mir_649,P2RX6P,LOC400891* | 2 | gain | 0.055 |
| 22 | 23905450 | 23953204 | 47755 | *IGLL1,C22orf43,DQ586720* | 1 | gain | 0.235 |
| 22 | 39399300 | 39430443 | 31144 | *APOBEC3C,APOBEC3D* | 1 | gain | 0.235 |
| 22 | 42955776 | 42966416 | 10641 | *SERHL2,RRP7B* | 1 | gain | 0.235 |
| 22 | 44234854 | 44563293 | 328440 | *SULT4A1,PNPLA5,PNPLA3,SAMM50,AX747952,PARVB,TRNA_SeC* | 1 | gain | 0.235 |
| 22 | 49388701 | 51188494 | 1799794 | *BC033837,C22orf34,BRD1,LOC90834,ZBED4,ALG12,CRELD2,PIM3,IL17REL,MLC1,MOV10L1,PANX2,TRABD,SELO,TUBGCP6,HDAC10,MAPK12,MAPK11,PLXNB2,DENND6B,PPP6R2,AB372727,SBF1,ADM2,MIOX,LMF2,NCAPH2,SCO2,TYMP,ODF3B,KLHDC7B,SYCE3,CPT1B,CHKB-CPT1B,BC048192,CHKB,CHKB-AS1,MAPK8IP2,ARSA,SHANK3,BC050343,ACR* | 1 | gain | 0.235 |
| 22 | 19024794 | 20470598 | 1445805 | *DGCR2,Y_RNA,DGCR11,DGCR14,TSSK2,GSC2,SLC25A1,CLTCL1,HIRA,MRPL40,C22orf39,BX648073,UFD1L,U84523,CDC45,CLDN5,LINC00895,SEPT5-GP1BB,TBX1,GNB1L,C22orf29,TXNRD2,COMT,MIR4761,ARVCF,TANGO2,MIR185,DGCR8,MIR3618,MIR1306,TRMT2A,RANBP1,ZDHHC8,LOC388849,LOC284865,LINC00896,RTN4R,MIR1286,KIAA1653,DGCR6L,LOC729444,TMEM191B,PI4KAP1,HV593096,HV593134,HV593127,HV593178,HV593135,7SK,RIMBP3,Metazoa_SRP* | 1 | loss | 0.235 |
| 22 | 21465835 | 21611337 | 145503 | *BCRP2,AL117485,POM121L7,DQ570150,BC039313,AK128837,GGT2* | 1 | loss | 0.235 |
| 22 | 21917141 | 22307519 | 390379 | *UBE2L3,YDJC,CCDC116,SDF2L1,MIR301B,MIR130B,PPIL2,YPEL1,MAPK1,PPM1F* | 1 | loss | 0.235 |
| 22 | 24396802 | 24404830 | 8029 | *GSTTP2* | 4 | loss | 0.003 |
| 22 | 33749276 | 34091543 | 342268 | *LARGE,MIR4764* | 1 | loss | 0.235 |
| 22 | 44074183 | 44112845 | 38663 | *EFCAB6* | 1 | loss | 0.235 |
| 22 | 46102643 | 46271595 | 168953 | *ATXN10,MIR4762* | 1 | loss | 0.235 |
| 22 | 51087264 | 51234443 | 147180 | *SHANK3,BC050343,ACR,RPL23AP82,RABL2B* | 2 | loss | 0.055 |

1. Six well known ASD-associated CNV loci

| **CNVs locus** | **CN segment (start)** | | **CN segment (stop)** | | **Size**  **(bp)** | | **Gene** | | **Case**  **(n=335)** | | **CNVs**  **type** | |
| --- | --- | --- | --- | --- | --- | --- | --- | --- | --- | --- | --- | --- |
| 1p11.2-q21.1 | 142569069 | 144007037 | | 1437969 | | *DQ579288,DQ586768,DQ583161,DQ590589,ANKRD20A12P,BC071797,DQ590126,DQ592442,BC053679,BC029473,AK056396,CR936796,DQ587539,DQ596206,BC070106,TRNA_Asn,DQ571491,LOC100130000,LINC00875,PPIAL4G,FAM72D,SRGAP2B* | | 2 | | gain | |  |
| 1q21.1 | 144991278 | 145290292 | | 299014 | | *LOC653513,PDE4DIP,BC065231,BX647792,SEC22B,NOTCH2NL,NBPF10,LOC100288142,NBPF9* | | 2 | | gain | |  |
| 1q21.1 | 145605318 | 145625694 | | 20376 | | *NBPF10,LOC100288142,RNF115,POLR3C* | | 1 | | loss | |  |
| 15q13.1 | 28709280 | 29073541 | | 364262 | | *MIR4509-1,GOLGA8G,JB175342,DQ578700,GOLGA8F,DQ588687,DQ599733,GOLGA6L1,DQ600136,DQ579907,LOC283767,DQ582071,AK311660,AK309255,AK307870,DQ593342,HERC2P9,DQ596685,DQ582448,DQ593032,DQ582939,DQ597560,WHAMMP2,LOC100289656,LOC646278* | | 2 | | gain | |  |
| 15q13.3 | 33145711 | 33546098 | | 400388 | | *FMN1,SNORD77,TMCO5B* | | 1 | | gain | |  |
| 16p11.2 | 33864347 | 34011271 | | 146924 | | *JB158072,LINC00273,JB175072* | | 2 | | gain | |  |
| 16p11.2 | 29591757 | 30191895 | | 600139 | | *LOC440354,SLC7A5P1,SPN,QPRT,C16orf54,BC041466,ZG16,KIF22,MAZ,AB209061,AK097472,PRRT2,BC029255,PAGR1,MVP,CDIPT,CDIPT-AS1,SEZ6L2,ASPHD1,KCTD13,TMEM219,TAOK2,HIRIP3,INO80E,DOC2A,C16orf92,FAM57B,AK097527,ALDOA,PPP4C,TBX6,YPEL3,AK097453,GDPD3,MAPK3,BOLA2* | | 1 | | gain | |  |
| 16p11.2 | 34197339 | 34503222 | | 305884 | | *UBE2MP1* | | 1 | | gain | |  |
| 16p11.2 | 31560514 | 31656578 | | 96064 | | *YBX3P1* | | 1 | | loss | |  |
| 22q11.21 | 18640300 | 18672873 | | 32574 | | *USP18,AK129567* | | 1 | | gain | |  |
| 22q11.21 | 19573160 | 21442671 | | 1869512 | | *SEPT5-GP1BB,TBX1,GNB1L,C22orf29,TXNRD2,COMT,MIR4761,ARVCF,TANGO2,MIR185,DGCR8,MIR3618,MIR1306,TRMT2A,RANBP1,ZDHHC8,LOC388849,LOC284865,LINC00896,RTN4R,MIR1286,KIAA1653,DGCR6L,LOC729444,TMEM191B,PI4KAP1,HV593096,HV593134,HV593127,HV593178,HV593135,7SK,RIMBP3,Metazoa_SRP,AK129567,AK302545,JX456220,USP41,ZNF74,SCARF2,KLHL22,MED15,BC035867,POM121L4P,DQ571461,TMEM191A,PI4KA,HV593183,HV593110,SERPIND1,SNAP29,CRKL,BC033281,BC127858,AIFM3,LZTR1,THAP7,DQ574263,THAP7-AS1,TUBA3FP,P2RX6,SLC7A4,Mir_649,P2RX6P,LOC400891* | | 2 | | gain | |  |
| 22q11.21 | 19024794 | 20470598 | | 1445805 | | *DGCR2,Y_RNA,DGCR11,DGCR14,TSSK2,GSC2,SLC25A1,CLTCL1,HIRA,MRPL40,C22orf39,BX648073,UFD1L,U84523,CDC45,CLDN5,LINC00895,SEPT5-GP1BB,TBX1,GNB1L,C22orf29,TXNRD2,COMT,MIR4761,ARVCF,TANGO2,MIR185,DGCR8,MIR3618,MIR1306,TRMT2A,RANBP1,ZDHHC8,LOC388849,LOC284865,LINC00896,RTN4R,MIR1286,KIAA1653,DGCR6L,LOC729444,TMEM191B,PI4KAP1,HV593096,HV593134,HV593127,HV593178,HV593135,7SK,RIMBP3,Metazoa_SRP* | | 1 | | loss | |  |
| 22q11.21 | 19006984 | 19568989 | | 562006 | | *DGCR5,DGCR9,DGCR10,DGCR2,Y_RNA,DGCR11,DGCR14,TSSK2,GSC2,SLC25A1,CLTCL1,HIRA,MRPL40,C22orf39,BX648073,UFD1L,U84523,CDC45,CLDN5,LINC00895* | | 1 | | gain | |  |
| 22q11.21 | 21465835 | 21611337 | | 145503 | | *BCRP2,AL117485,POM121L7,DQ570150,BC039313,AK128837,GGT2* | | 1 | | loss | |  |
| 22q11.21-q11.22 | 21917141 | 22307519 | | 390379 | | *UBE2L3,YDJC,CCDC116,SDF2L1,MIR301B,MIR130B,PPIL2,YPEL1,MAPK1,PPM1F* | | 1 | | loss | |  |
| 22q13.32-q13.33 | 49388701 | 51188494 | | 1799794 | | *BC033837,C22orf34,BRD1,LOC90834,ZBED4,ALG12,CRELD2,PIM3,IL17REL,MLC1,MOV10L1,PANX2,TRABD,SELO,TUBGCP6,HDAC10,MAPK12,MAPK11,PLXNB2,DENND6B,PPP6R2,AB372727,SBF1,ADM2,MIOX,LMF2,NCAPH2,SCO2,TYMP,ODF3B,KLHDC7B,SYCE3,CPT1B,CHKB-CPT1B,BC048192,CHKB,CHKB-AS1,MAPK8IP2,ARSA,SHANK3,BC050343,ACR* | | 1 | | gain | |  |
| 22q13.33 | 51087264 | 51234443 | | 147180 | | *SHANK3,BC050343,ACR,RPL23AP82,RABL2B* | | 2 | | loss | |  |

1. Summary of CNV findings at the *PARK2* locus

|  | **Initiation ^b^** | | **Replication ^c^** | | | **Combination ^d^** | | |
| --- | --- | --- | --- | --- | --- | --- | --- | --- |
|  | **ASD case** | **Healthy control** | | **ASD case** | **Healthy control** | | **ASD case** | **Healthy control** |
| **Total, n** | 335 | 1093 | | 301 | 301 | | 636 | 1394 |
| **Sex**  Male, n (%) | 299 (89) | 525 (48) | | 266 (88) | 193 (64) | | 565 (89) | 718 (52) |
| Female, n (%) | 36 (11) | 568 (52) | | 35 (12) | 108 (36) | | 71 (11) | 676 (48) |
| **Age, mean (sd; years)** | 9.18 (4.33) | 68.07 (10.12) | | 11.25 (5.47) | 19.59 (8.90) | | 10.17 (5.01) | 57.60 (22.26) |
| **CNV, total ^a^** | 4 | 2 | | 2 | 0 | | 6 | 2 |
| **A**- chr6: 162590018-162840211 | 1 (1 dup) | 2 (2 dup) | | 2 (1 dup, 1 del) | 0 | | 3 (2 dup, 1 del) | 2 (2 dup) |
| **B**- chr6: 162451920-162507690 | 2 (2 del) | 0 | | 0 | 0 | | 2 (2 del) | 0 |
| **C**- chr6: 162187125-162402914 | 1 (1 del) | 0 | | 0 | 0 | | 1 (1 del) | 0 |

Abbreviations: ASD, autism spectrum disorder; CNV, copy number variation; dup, duplication; del, deletion.

^a^ chr6:162,187,125-162,840,211. (653 kb)

**^b^** Fisher’s exact 2-sided P-value (case vs. control) *P* _total CNV of initiation_ = 0.030*

**^c^** Fisher’s exact 2-sided P-value (case vs. control) *P* _total CNV of replication_ = 0.499

**^d^** Fisher’s exact 2-sided P-value (case vs. control) *P* _total CNV of combination_ = 0.014*

1. Autism-Spectrum Quotient and Adult Self-Report Inventory-IV

| Proband | Father | | | | | | | Mather | | | | | | |
| --- | --- | --- | --- | --- | --- | --- | --- | --- | --- | --- | --- | --- | --- | --- |
|  | AQ1 | AQ2 | AQ3 | AQ4 | AQ5 | AQ Total score | ASRI | AQ1 | AQ2 | AQ3 | AQ4 | AQ5 | AQ Total score | ASRI |
| U1469 | 6 | 4 | 6 | 4 | 7 | 27 | - | 1 | 4 | 5 | 1 | 3 | 14 | - |
| U1290 | 5 | 2 | 0 | 3 | 4 | 14 | - | 7 | 3 | 4 | 3 | 5 | 22 | - |
| U1859 | 7 | 6 | 1 | 4 | 5 | 23 | - | 7 | 6 | 4 | 3 | 5 | 25 | - |
| U1984 | 1 | 4 | 2 | 1 | 3 | 11 | - | 3 | 3 | 5 | 0 | 3 | 14 | ANX |
| U2650 | 1 | 5 | 6 | 0 | 3 | 15 | - | 2 | 3 | 0 | 2 | 3 | 10 | ANX, OCD, SLED |
| U2890 | - | - | - | - | - | - | - | - | - | - | - | - | - | - |

**Autism-Spectrum Quotient (AQ) (**50 items, total scores can range from 0 to 50), **AQ1:** Social skill (score 0-10), **AQ2:** Attention switching (score 0-10), **AQ3:** Attention to detail (score 0-10), **AQ4:** Communication (score 0-10), **AQ5:** Imagination (score 0-10), **AQ cut-off score** is 32.

**ASRI :** Adult Self*-*Report Inventory-IV **, ANX:** anxiety disorders, **OCD:** obsessive–compulsive disorder, **SLED:** sleep disorders.

**Family 5 (case age >10 years)**

| **ID** | Social skill | Attention switching | Attention to detail | Communication | Imagination | **AQ Total score** |
| --- | --- | --- | --- | --- | --- | --- |
| Proband (U2650) | 6 | 5 | 3 | 5 | 6 | **25** |
| Father | 1 | 5 | 6 | 0 | 3 | **15** |
| Mother | 2 | 3 | 0 | 2 | 3 | **10** |
| Sister | 2 | 4 | 6 | 1 | 2 | **15** |

1. SUPPLEMENTARY REFERENCES

1. Girirajan S, Johnson RL, Tassone F, Balciuniene J, Katiyar N, Fox K, Baker C, Srikanth A, Yeoh KH, Khoo SJ, Nauth TB, Hansen R, Ritchie M, Hertz-Picciotto I, Eichler EE, Pessah IN, Selleck SB: **Global increases in both common and rare copy number load associated with autism.** *Hum Mol Genet* 2013, **22:**2870-2880.

2. Lord C, Rutter M, Le Couteur A: **Autism Diagnostic Interview-Revised: a revised version of a diagnostic interview for caregivers of individuals with possible pervasive developmental disorders.** *J Autism Dev Disord* 1994, **24:**659-685.

3. Chien YL, Wu YY, Chen CH, Gau SS, Huang YS, Chien WH, Hu FC, Chao YL: **Association of HLA-DRB1 alleles and neuropsychological function in autism.** *Psychiatr Genet* 2012, **22:**46-49.

4. Chien YL, Wu YY, Chiu YN, Liu SK, Tsai WC, Lin PI, Chen CH, Gau SS, Chien WH: **Association study of the CNS patterning genes and autism in Han Chinese in Taiwan.** *Prog Neuropsychopharmacol Biol Psychiatry* 2011, **35:**1512-1517.

5. Gau SS, Liao HM, Hong CC, Chien WH, Chen CH: **Identification of two inherited copy number variants in a male with autism supports two-hit and compound heterozygosity models of autism.** *Am J Med Genet B Neuropsychiatr Genet* 2012, **159B:**710-717.

6. Lin PI, Chien YL, Wu YY, Chen CH, Gau SS, Huang YS, Liu SK, Tsai WC, Chiu YN: **The WNT2 gene polymorphism associated with speech delay inherent to autism.** *Res Dev Disabil* 2012, **33:**1533-1540.

7. Constantino JN, Todd RD: **Genetic structure of reciprocal social behavior.** *Am J Psychiatry* 2000, **157:**2043-2045.

8. Constantino JN, Davis SA, Todd RD, Schindler MK, Gross MM, Brophy SL, Metzger LM, Shoushtari CS, Splinter R, Reich W: **Validation of a brief quantitative measure of autistic traits: comparison of the social responsiveness scale with the autism diagnostic interview-revised.** *J Autism Dev Disord* 2003, **33:**427-433.

9. Gau SS, Liu LT, Wu YY, Chiu YN, Tsai WC: **Psychometric properties of the Chinese version of the social responsiveness scale.** *Research in Autism Spectrum Disorders* 2013, **7:**349-360.

10. Constantino JN, Przybeck T, Friesen D, Todd RD: **Reciprocal social behavior in children with and without pervasive developmental disorders.** *J Dev Behav Pediatr* 2000, **21:**2-11.

11. Allen SR, Thorndike RM: **Stability of the WAIS-R and WISC-III factor structure using cross-validation of covariance structures.** *J Clin Psychol* 1995, **51:**648-657.

12. Pascualvaca DM, Fantie BD, Papageorgiou M, Mirsky AF: **Attentional capacities in children with autism: is there a general deficit in shifting focus?** *J Autism Dev Disord* 1998, **28:**467-478.

13. Liss M, Fein D, Allen D, Dunn M, Feinstein C, Morris R, Waterhouse L, Rapin I: **Executive functioning in high-functioning children with autism.** *J Child Psychol Psychiatry* 2001, **42:**261-270.

14. Shu BC, Lung FW, Tien AY, Chen BC: **Executive function deficits in non-retarded autistic children.** *Autism* 2001, **5:**165-174.
